# Bayesian Inference for Spatially-Temporally Misaligned Data Using Predictive Stacking

**DOI:** 10.1002/env.70072

**Published:** 2026-01-25

**Authors:** Soumyakanti Pan, Sudipto Banerjee

**Affiliations:** Department of Biostatistics, University of California Los Angeles, Los Angeles, California, USA

**Keywords:** change of support, model combination, modular inference, weak identifiability

## Abstract

Air pollution remains a major environmental risk factor that is often associated with adverse health outcomes. However, quantifying and evaluating its effects on human health is challenging due to the complex nature of exposure data. Recent technological advances have led to the collection of various indicators of air pollution at increasingly high spatial-temporal resolutions (e.g., daily averages of pollutant levels at spatial locations referenced by latitude-longitude). However, health outcomes are typically aggregated over several spatial-temporal coordinates (e.g., annual prevalence for a county) to comply with survey regulations. This article develops a Bayesian hierarchical model to analyze such spatially-temporally misaligned exposure and health outcome data. We develop Bayesian predictive stacking for spatially and temporally misaligned data to optimally combine inference from multiple predictive spatial-temporal models. Stacking allows us to avoid iterative estimation algorithms such as Markov chain Monte Carlo that struggle due to convergence issues inflicted by the presence of weakly identified parameters. We apply our proposed method to study the effects of ozone on asthma in the state of California.

## 1 | Introduction

Spatial and temporal misalignment refers to the setting in which different variables are observed over incompatible spatial supports and/or at asynchronous time points or intervals. To be more specific, spatial misalignment arises when variables are measured at different spatial resolutions, that is, different sets of locations or areas. For example, a variable might be observed at points (e.g., air quality monitoring stations), which we call *point-referenced* data, while another is aggregated over administrative units (e.g., counties or census tracts), which we call *block* data. Similarly, temporal misalignment occurs when variables are recorded on different time scales.

In this article, we devise a Bayesian hierarchical modeling framework to study the effects of ozone on asthma-related health emergencies among California residents. Several studies collectively underscore the significant impact of exposure to ozone on asthma-related emergency department visits (e.g., Gharibi et al. 2019; Nassikas et al. 2020). We obtain data on the monthly average concentration of ozone in California measured by air quality monitoring stations located at different locations throughout the state. Thus, the ozone measurements are spatially point-referenced and temporally aggregated at a monthly scale. However, data on asthma-related emergency department visits among Californian residents are reported at the county level and aggregated annually. We refer to such disparity in both spatial and temporal resolutions as spatial-temporal misalignment.

Spatially-temporally misaligned data presents significant challenges for coherent modeling, prediction, and inference. This problem is well known as the *change of support* and *modifiable areal unit* problem (Cressie 1996; Mugglin et al. 2000; Gelfand et al. 2001; Gotway and Young 2002). Most of the literature focuses on estimating variables at unobserved spatial resolutions or integrating data across varying spatial scales (Banerjee et al. 2025; Zhong and Moraga 2023). However, efforts to estimate the association between an outcome of interest and a spatially-temporally misaligned covariate remain rather limited (Zhu et al. 2003; Cameletti et al. 2019), with existing approaches typically overlooking temporal misalignment. Moreover, a key challenge in analyzing spatial-temporal exposure data is missing observations resulting from intermittent monitoring or data removal due to quality issues. Traditional approaches often use imputation methods that aim to reconstruct the complete data set (e.g., Zhu et al. 2003; Quick et al. 2013). Our approach forgoes imputation and works directly with the available irregularly spaced data to produce a more robust framework for spatial-temporal analysis.

We contribute in two novel aspects. First, we propose a modular Bayesian inference framework (Bayarri et al. 2009; Jacob et al. 2017) that regresses an outcome on a spatially-temporally misaligned covariate, based on noisy observations of the latter, ensuring fully model-based propagation of inferential uncertainty. Second, we develop predictive stacking for estimation of such models that analyze spatially-temporally misaligned data, thus representing a methodological advancement over previous work that considered spatial Gaussian data (Zhang et al. 2025; Wakayama and Banerjee 2024; Presicce and Banerjee 2025), and spatial-temporal non-Gaussian data (Pan et al. 2026). Stacking (Wolpert 1992; Breiman 1996; Clyde and Iversen 2013) is used in machine learning as an effective alternative (Le and Clarke 2017; Yao et al. 2018) to traditional Bayesian model averaging (Madigan et al. 1996; Hoeting et al. 1999). The underlying idea in predictive stacking is to optimally assimilate posterior distributions on a grid of candidate values corresponding to intractable and weakly identified hyperparameters, such as spatial-temporal decay, smoothness, and noise-to-spatial-temporal variance ratio (Zhang 2004; Zhang and Zimmerman 2005; Tang et al. 2021), which impede the convergence of iterative algorithms such as Markov chain Monte Carlo (MCMC). Stacking also differs from quadrature-based approaches, such as INLA (Rue et al. 2009), in that we avoid approximating the posterior distribution of weakly identified parameters. Instead, we average, or “stack”, individual posterior distributions, using weights obtained by optimizing a proper scoring rule (Gneiting and Raftery 2007).

The remainder of the article is structured as follows. Section 2 describes the data set that motivates our methodology. Section 3 introduces our Bayesian hierarchical model for analyzing the spatially-temporally misaligned data and states key assumptions critical for posterior inference. Section 4 develops predictive stacking and a computationally efficient algorithm for model estimation. Section 5 reports results from different simulation experiments. Section 6 presents our data analysis, while Section 7 concludes with a brief discussion.

## 2 | Data

**FIGURE 1 |:**
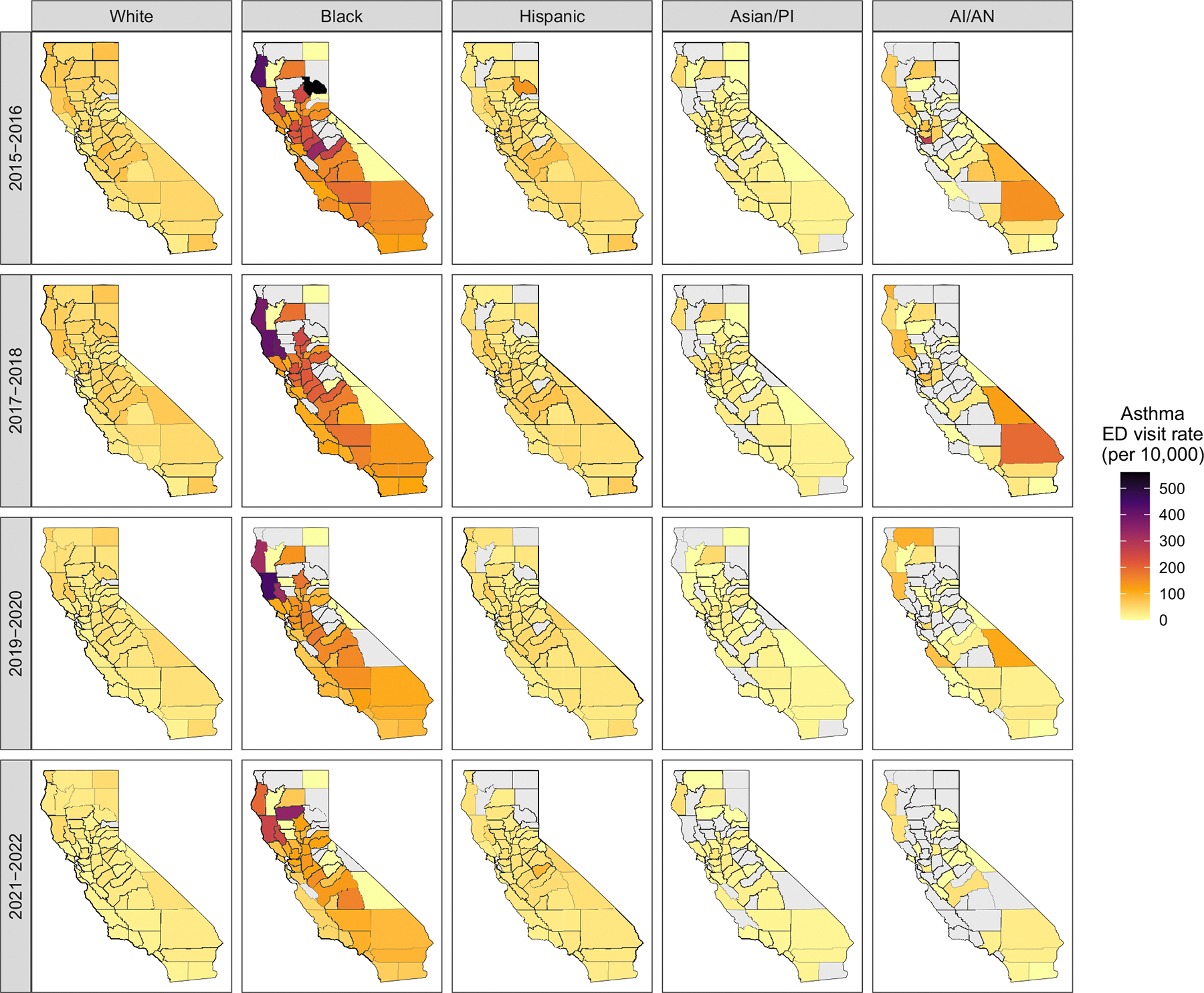
Biennial average asthma-related emergency department visit rates (per 10,000) by racial group for each California county from 2015 through 2022. For visualization, rates are averaged over consecutive 2-year periods.

The data set on adverse health outcomes comprises annual county-level rates of visits to the emergency department (ED) related to asthma per 10,000 residents of California. These data are obtained from the California Department of Public Health (CDPH) and are derived from the Emergency Department database of the Department of Access and Information to Health Care, which includes records from all licensed hospitals in the state (California Breathing Asthma Program 2024). We analyze data consisting of age-adjusted rates stratified by race/ethnicity (white, black, Hispanic, Asian/Pacific Islander, American Indian/Alaskan Native), derived from annual aggregated counts of asthma-related ED visits from 2015 to 2022 for each of the 58 counties in California (see Figure S1 in the Supporting Information). ED visit counts are based only on primary discharge diagnosis codes. The database omits rates based on (1) counts < 12 due to statistical instability, and (2) counts ≤ 5 according to the CalHHS Data De-identification Guidelines. This results in approximately 35% missing data that mainly affect the Asian/Pacific Islander and American Indian/Alaskan Native groups. Figure 1 maps the aggregated 2-year rates of asthma-related emergency department visits observed for each county in California during 2015–2022, revealing a clear pattern of racial disparity, with some counties consistently showing high rates. The age-adjusted rates are highly positive skewed, ranging from 0 to 560.9 per 10,000 residents, with the majority between 15 and 60 (see Figure S3 in the Supporting Information). Hence, we analyze log-transformed rates, as they are more amenable to Gaussian assumptions that yield closed-form posteriors, enabling inference without iterative algorithms.

**FIGURE 2 |:**
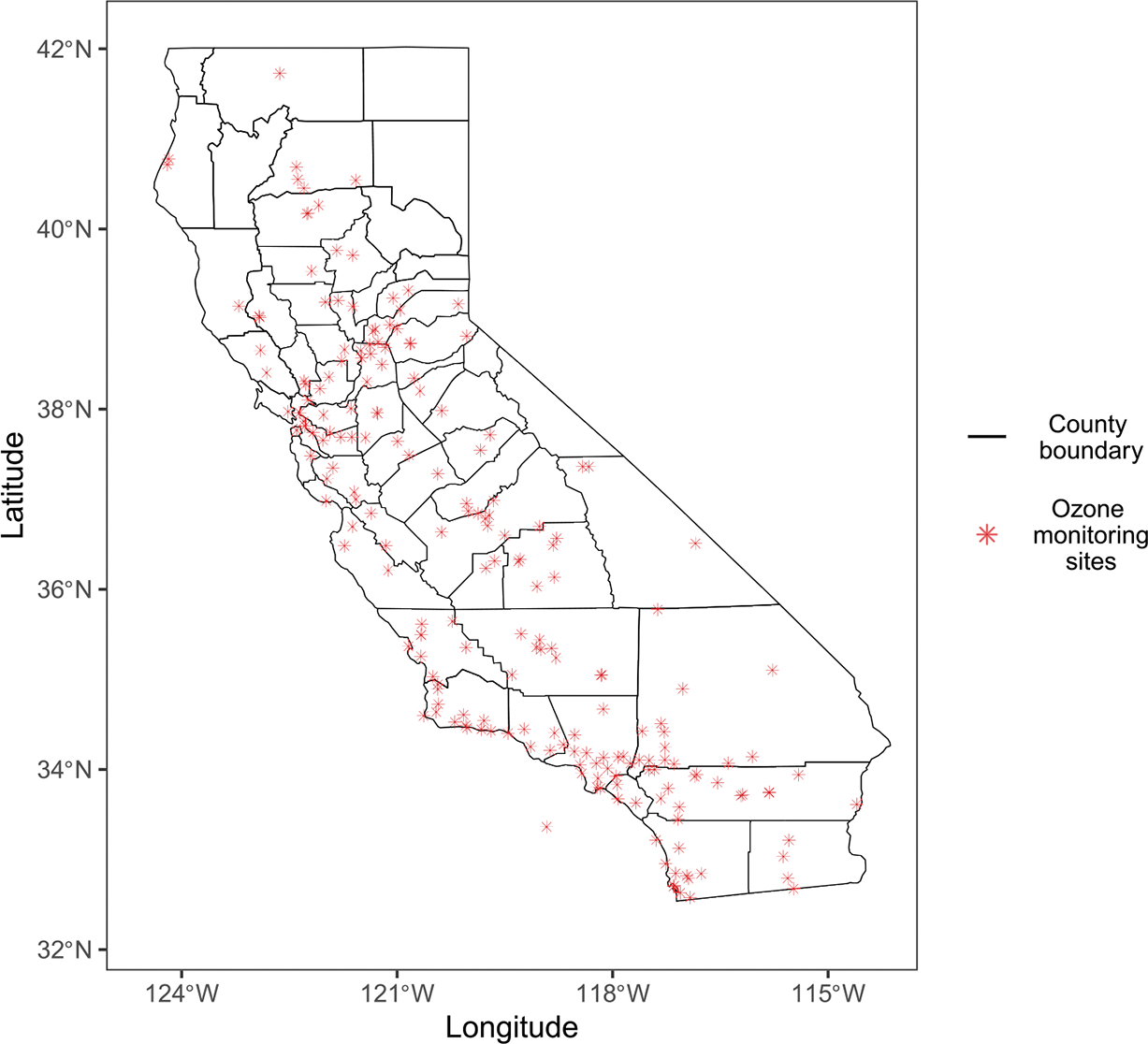
County boundaries of California and the geographic locations of 200 ozone monitoring sites active from 2015 to 2022.

For exposure data, we extracted hourly measurements of ozone concentration (in parts per million) from the California Air Quality and Meteorological Information System (AQMIS) database of the California Air Resources Board for the years 2015–2022. These data were recorded from about 200 air quality monitoring sites in California that were active during this time period. Figure 2 maps the geographic locations of these monitoring sites located within the county boundaries of California, highlighting the heterogeneous spatial distribution of air quality surveillance in counties.

**FIGURE 3 |:**
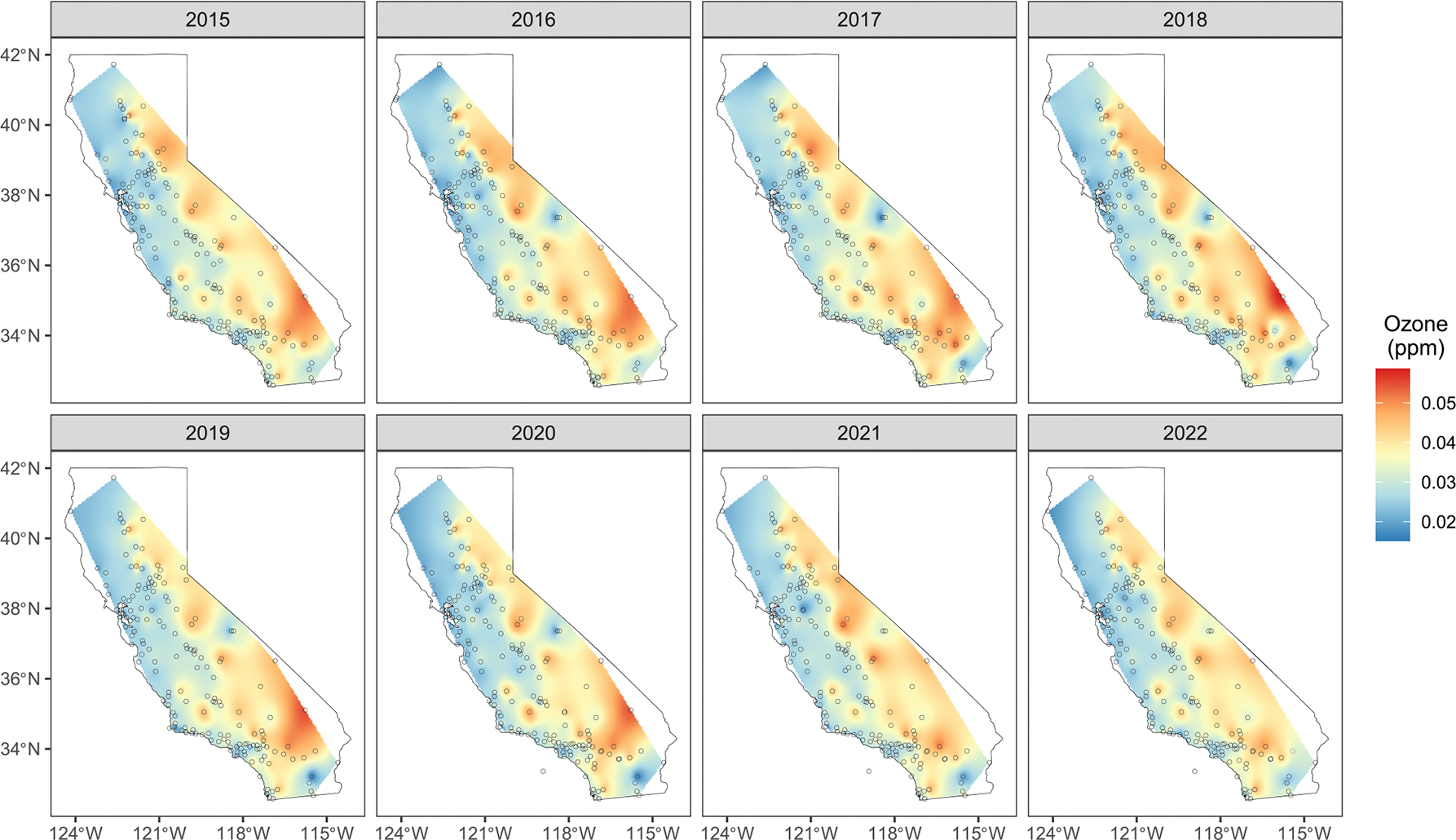
Interpolated spatial surface of annual (summed over months) ozone concentration (in parts per million) for California from 2015 to 2022. The geographic coordinates of the air quality monitoring stations are marked by black circles.

The figure also reveals a clear regional clustering of sites near urban and coastal areas in central and southern California, with notable sparsity in the eastern rural inland regions. For our analysis, we aggregate hourly data into monthly average ozone concentrations. As not all monitoring sites were active during the study period and have missing monthly records, the resulting data set is temporally unbalanced between sites. In total, the data set comprises measurements of ozone concentrations at 15,725 unique spatial-temporal locations, where the temporal component is referenced by monthly intervals rather than exact timestamps. Figure 3 features interpolated spatial surfaces of annual average ozone concentration between 2015 and 2022, showing a clear spatial pattern, with the sparsely sampled eastern inland regions recording higher ozone concentrations, demonstrating spatial imbalances in monitoring coverage.

## 3 | Bayesian Spatial-Temporal Hierarchical Model

### 3.1 | Multi-Resolution Spatial-Temporal Process

We consider a spatial-temporal process as an uncountable set of random variables, say {Z(ℓ):ℓ∈𝒟}, which is endowed with a probability law specifying the joint distribution for any finite sample of locations in 𝒟=𝒮×𝒯, where $𝒮\subset R^{2}$ and 𝒯⊂[0,∞) are space and time domains, respectively, and ℓ=(s,t) is a spacetime coordinate with s∈𝒮 and t∈𝒯 (see, e.g., Gneiting and Guttorp 2010). Subsequently, we define two new types of space-time coordinates $\tilde{\ell }=(\tilde{s},\tilde{I})$ and L=(B,I), where $\tilde{s}\in 𝒮,\;B\subset 𝒮$ denotes a block or a region within 𝒮, and $I,\;\tilde{I}\subset 𝒯$ denote intervals in 𝒯. We extend Z(ℓ) to these new coordinate systems as $\left\{Z(\tilde{\ell }):\tilde{\ell }\in 𝒮\times B(𝒯)\right\}$ and {Z(L):L∈B(𝒮×𝒯)}, where B(A) denotes the collection of Borel-measurable subsets of a set A (Billingsley 1995), and

(1) $$\begin{array}{l} Z(\tilde{\ell })=Z(\tilde{s},\tilde{I})={\left|\tilde{I}\right|}^{-1}\int_{\tilde{I}}Z\left(\tilde{s},t\right)dt\;,\\ Z(L)=Z(B,I)=(\left|B\right|\left|I\right|{)}^{-1}\int_{I}\int_{B}Z\left(s,t\right)dsdt\;, \end{array}$$

where $|B|=\int_{B}1ds$ denotes the area of B, and $|I|=\int_{I}1dt$, $|\tilde{I}|=\int_{\tilde{I}}1dt$ denote the lengths of the intervals I and $\tilde{I}$, respectively. This implies that the random variable $Z(\tilde{\ell })$ denotes the realization of the stochastic process obtained by a temporal averaging of Z(ℓ) over the interval $\tilde{I}$ at location $\tilde{s}$. In our context, this corresponds to the monthly average concentration of ozone at the location $\tilde{s}$, averaged over the month $\tilde{I}$. Similarly, Z(L) is a realization from the stochastic process obtained by averaging Z(ℓ) over a spatial-temporal block L, which includes averaging spatially over the region B and averaging temporally over the interval I. This may represent ozone concentrations in the county defined by the spatial block B, averaged over the annual interval I.

A stationary Gaussian process specification for Z(ℓ) enables us to analytically derive a joint distribution for the spatial-temporal process at each resolution. To be specific, suppose Z(ℓ) is a Gaussian process with mean function μ(ℓ;γ) and covariance function $\sigma^{2}C(\ell ,\ell^{\prime };\phi )$, where μ(⋅;γ) denotes a trend surface with coefficient vector $\gamma ,\;\sigma^{2}$ denotes spatial-temporal variance, and ϕ denotes a generic parameter vector characterizing spatial-temporal decay or smoothness. We collectively call ϕ as “process parameters”. For any given spatial-temporal coordinates $\left({\tilde{\ell }}_{j}=({\tilde{s}}_{j},{\tilde{I}}_{j}):j=1,\;\ldots ,N\right\}$ and $\left\{L_{k}=\left(B_{k},I_{k}\right):k=\right.1,\;\ldots ,K\}$, define the N×1 vector $Z_{\tilde{\ell }}={\left(Z\left({\tilde{\ell }}_{1}\right),\;\ldots ,Z\left({\tilde{\ell }}_{N}\right)\right)}^{⊤}$ and the K×1 vector $Z_{L}={\left(Z\left(L_{1}\right),\;\ldots ,Z\left(L_{K}\right)\right)}^{⊤}$. Then, we have

(2) pZℓ˜ZLγ,σ2,ϕ=NZℓ˜ZLμℓ˜(γ)μL(γ),σ2Cℓ˜(ϕ)Cℓ˜,L(ϕ)Cℓ˜,L(ϕ)⊤CL(ϕ),

where

(3) $$\begin{array}{ll} {\left(\mu_{\tilde{\ell }}(\gamma )\right)}_{j} & ={\left|{\tilde{I}}_{j}\right|}^{-1}\int_{{\tilde{I}}_{j}}\mu (\left({\tilde{s}}_{j},t\right);\gamma )dt,\\ {\left(\mu_{L}(\gamma )\right)}_{k} & ={\left(\left|I_{k}\right|\left|B_{k}\right|\right)}^{-1}\int_{L_{k}}\mu (\ell ;\gamma )d\ell \;,\\ {\left(C_{\tilde{\ell }}(\phi )\right)}_{j,j^{\prime }} & ={\left(\left|{\tilde{I}}_{j}\right|\left|{\tilde{I}}_{j^{\prime }}\right|\right)}^{-1}\int_{{\tilde{I}}_{j^{\prime }}}\int_{{\tilde{I}}_{j}}C\left(\left({\tilde{s}}_{j},t\right),\left({\tilde{s}}_{j^{\prime }},t^{\prime }\right);\phi \right)dtdt^{\prime },\;\\ {\left(C_{\tilde{\ell },L}(\phi )\right)}_{j,k} & ={\left(\left|{\tilde{I}}_{j}\right|\left|I_{k}\right|\left|B_{k}\right|\right)}^{-1}\int_{{\tilde{I}}_{j}}\int_{I_{k}}\int_{B_{k}}C\left(\left({\tilde{s}}_{j},t^{\prime }\right),(s,t);\phi \right)dsdtdt^{\prime },\;\\ {\left(C_{L}(\phi )\right)}_{k,k^{\prime }} & ={\left(\left|L_{k^{\prime }}\right|\left|L_{k}\right|\right)}^{-1}\int_{L_{k^{\prime }}}\int_{L_{k}}C\left(\ell ,\ell^{\prime };\phi \right)d\ell d\ell^{\prime }\;, \end{array}$$

for $j,\;j^{\prime }=1,\;\ldots ,N,\;k,\;k^{\prime }=1,\;\ldots ,K$, and $\left|L_{k}\right|=\left|B_{k}\right|\left|I_{k}\right|$ for each k. The joint distribution (2) provides a unified framework that connects the process across the two spatial-temporal resolutions, enabling tractable posterior predictive inference in spatial-temporal blocks $L_{k}$ for each k, conditional on observed realizations in point-referenced temporal blocks ${\tilde{\ell }}_{j},\;j=1,\;\ldots ,N$.

### 3.2 | Conjugate Bayesian Hierarchical Model

Let $ℒ=\left\{L_{k}:k=1,\;\ldots ,K\right\}$ be a fixed set of K spatial-temporal blocks in B(𝒮×𝒯), where $L_{k}$ is of the form ($B_{k},I_{k}$) with $B_{k}$ being a block or region in 𝒮 and $I_{k}$ an interval in 𝒯, for each k. Let $Y(ℒ)={\left(Y\left(L_{1}\right),\;\ldots ,Y\left(L_{k}\right)\right)}^{⊤}$, which we simply denote by Y, be the K×1 vector of outcomes observed at ℒ. The key challenge of our model is that we do not observe the covariates that are assumed to explain the outcome at the same spatial-temporal resolution at which Y is observed. Suppose $\tilde{ℒ}=\left\{{\tilde{\ell }}_{j}:j=1,\;\ldots ,N\right\}$, where ${\tilde{\ell }}_{j}$ is of the form $\left({\tilde{s}}_{j},{\tilde{I}}_{j}\right)$ for each j, be the N spatial-temporal coordinates in 𝒮×B(𝒯), at which we observe the covariate $X(\tilde{ℒ})={\left(X\left({\tilde{\ell }}_{1}\right),\;\ldots ,X\left({\tilde{\ell }}_{N}\right)\right)}^{⊤}$, which we simply write as the N×1 vector X. We further assume that $X(\tilde{ℒ})$ are noisy measurements of a latent spatial-temporal process on 𝒮×B(𝒯) derived from a parent process Z(ℓ) on 𝒟 following the stochastic integral (1). This setup is particularly motivated by the asthma and ozone datasets described in Section 2, where the outcome is measured at the county level and aggregated annually, while the covariate is spatially referenced to points, but aggregated on a monthly scale.

We build a hierarchical model by jointly modeling both the outcome and the covariate as conditionally dependent on a shared latent spatial-temporal process,

(4) $$\begin{array}{ll} Y\left(L_{k}\right) & =w{\left(L_{k}\right)}^{⊤}\beta_{1}+\beta_{2}Z\left(L_{k}\right)+\epsilon_{k},\\ \epsilon_{k} & \overset{\text{ind}}{\sim }N\left(0,{\left(\left|B_{k}\right|\left|I_{k}\right|\right)}^{-1}\tau^{2}\right),\;k=1,\;\ldots ,K\;,\\ {\left(\beta_{1}^{⊤},\beta_{2}\right)}^{⊤}|\tau^{2} & \sim \;N\left(\mu_{\beta },\tau^{2}V_{\beta }\right),\;\tau^{2}\sim IG\left(a_{\tau },b_{\tau }\right)\;,\\ X\left({\tilde{\ell }}_{j}\right) & =Z\left({\tilde{\ell }}_{j}\right)+e_{j},\;e_{j}\overset{\text{ind}}{\sim }N(0,{\left|{\tilde{I}}_{j}\right|}^{-1}\delta^{2}\sigma^{2}),\;j=1,\;\ldots ,N\;,\\ Z(\ell )|\gamma ,\sigma^{2},\phi & \sim GP\left(\mu (\ell ;\gamma ),\sigma^{2}C(\cdot ,\cdot ;\phi )\right),\;\mu (\ell ;\gamma )=\psi (\ell {)}^{⊤}\gamma \;,\\ \gamma |\sigma^{2} & \sim \;N\left(\mu_{\gamma },\sigma^{2}V_{\gamma }\right),\;\sigma^{2}\sim IG\left(a_{\sigma },b_{\sigma }\right)\;, \end{array}$$

where $w\left(L_{k}\right)={\left(w_{1}\left(L_{k}\right),\;\ldots ,w_{p}\left(L_{k}\right)\right)}^{⊤}$ denotes a p×1 vector of confounders, $\beta_{1}$ is the corresponding p×1 vector of fixed effects, and, $\beta_{2}$ is a scalar and denotes the slope corresponding to the covariate $Z\left(L_{k}\right)$, which denotes the latent spatial-temporal process at $L_{k}$, for each k. Instead of $Z\left(L_{K}\right)$, we observe $X\left({\tilde{\ell }}_{j}\right)$, which are noisy observations of $Z\left({\tilde{\ell }}_{j}\right)$, which denotes the latent process at a different spatial-temporal resolution from $Z\left(L_{k}\right)$. Note that both $Z\left(L_{k}\right)$ for each k, and $Z\left({\tilde{\ell }}_{j}\right)$ for each j, are completely unobserved, and their joint probability law is specified by the assumption of a Gaussian process (GP) Z(ℓ) on 𝒟, following (2). The mean function of the GP is characterized by a r×1 basis $\psi (\ell )={\left(\psi_{1}(\ell ),\;\ldots ,\psi_{r}(\ell )\right)}^{⊤}$, and its corresponding r×1 slope vector γ. The purpose of ψ(ℓ) is to model a global trend in the spatial-temporal process, capturing the overall mean, seasonal variations, etc. The term $\epsilon_{k}$ denotes white noise defined only at the coordinates in ℒ, and captures the heteroskedasticity arising from inhomogeneity in the volumes of the spatial-temporal blocks at which the outcome is observed. Similarly, $e_{j}$ captures the measurement error for $X\left({\tilde{\ell }}_{j}\right)$ for each j, and are defined only at $\tilde{ℒ}$. It is crucial to note that, unlike Z(⋅), $\epsilon_{j}$ and $e_{k}$ are distinct white noise processes and do not originate from a parent white noise process on 𝒟. Here, $\delta^{2}$ is the noise-to-spatial-temporal variance ratio.

Let W be the K×p matrix with (k,u) th element $w_{k}\left(L_{u}\right)$ for k=1,…,K and u=1,…,p. Collect the regression coefficients into the (p+1)×1 vector $\beta ={\left(\beta_{1}^{⊤},\beta_{2}\right)}^{⊤}$. Given $\tau^{2}$, we place a multivariate Gaussian prior $N\left(\mu_{\beta },\tau^{2}V_{\beta }\right)$ on β, and subsequently place an inverse-gamma prior $IG\left(a_{\tau },b_{\tau }\right)$ on $\tau^{2}$. Similarly, we also place a multivariate Gaussian prior $N\left(\mu_{\gamma },\sigma^{2}V_{\gamma }\right)$ on γ conditional on $\sigma^{2}$, and then place an inverse-gamma prior $IG\left(a_{\sigma },b_{\sigma }\right)$ on $\sigma^{2}$. Define K×1 vector $Z_{L}={\left(Z\left(L_{1}\right),\;\ldots ,Z\left(L_{K}\right)\right)}^{⊤}$, and N×1 vector $Z_{\tilde{\ell }}={\left(Z\left({\tilde{\ell }}_{1}\right),\;\ldots ,Z\left({\tilde{\ell }}_{N}\right)\right)}^{⊤}$. Hence, we write the joint distribution of the data, latent process and the model parameters $p\left(Y,X,Z_{L},Z_{\tilde{𝒯}},\beta ,\gamma ,\tau^{2},\sigma^{2}\right)$ as

(5) $$\begin{array}{l} N(Y|W\beta_{1}+\beta_{2}Z_{L},\tau^{2}D_{L})\times N\left(\beta |\mu_{\beta },\tau^{2}V_{\beta }\right)\times IG\left(\tau^{2}|a_{\tau },b_{\tau }\right)\\ \;\times \;N\left(X|Z_{\tilde{\ell }},\delta^{2}\sigma^{2}D_{\tilde{\ell }}\right)\times N\left(Z_{\tilde{\ell }}|{\tilde{\Psi }}_{\gamma },\sigma^{2}C_{\tilde{\ell }}(\phi )\right)\\ \;\times \;N(Z_{L}|\mu_{L|\tilde{\ell }},\sigma^{2}C_{L|\tilde{\ell }})\times N\left(\gamma |\mu_{\gamma },\sigma^{2}V_{\gamma }\right)\times IG\left(\sigma^{2}|a_{\sigma },b_{\sigma }\right)\;, \end{array}$$

where $D_{L}$ is K×K diagonal matrix with the k th diagonal element ${\left(\left|B_{k}\right|\left|I_{k}\right|\right)}^{-1}$ for each k, and $D_{\tilde{\ell }}$ is N×N diagonal matrix with the j th diagonal element ${\left|{\tilde{I}}_{j}\right|}^{-1}$ for each j. Following (2), we have

(6) $$\begin{array}{l} \mu_{L|\tilde{\ell }}={\bar{\Psi }}_{\gamma }+C_{\tilde{\ell },L}(\phi {)}^{⊤}C_{\tilde{\ell }}^{-1}(\phi )\left(Z_{\tilde{\ell }}-{\tilde{\Psi }}_{\gamma }\right)\\ \;\text{and}\;C_{L|\tilde{\ell }}=C_{L}(\phi )-C_{\tilde{\ell },L}(\phi {)}^{⊤}C_{\tilde{\ell }}(\phi {)}^{-1}C_{\tilde{\ell },L}(\phi )\;, \end{array}$$

where N×N matrix $C_{\tilde{\ell }}(\phi )$ and N×K matrix $C_{\tilde{\ell },L}(\phi )$ are as defined in (3). Furthermore, the N×r basis matrix $\tilde{\Psi }$ is known and has (j,v)th element ${\tilde{\psi }}_{v}\left({\tilde{\ell }}_{j}\right)={\left|{\tilde{I}}_{j}\right|}^{-1}\int_{{\tilde{I}}_{j}}\psi_{v}(s,t)dt$ for v=1,…,r and j=1,…,N. Similarly, the K×r basis matrix $\bar{\Psi }$ is known, with (k,v)th element ${\bar{\psi }}_{v}\left(L_{k}\right)={\left(\left|L_{k}\right|\right)}^{-1}\int_{L_{k}}\psi (\ell )d\ell$ for k=1,…,K and v=1,…,r. Equation 5 factorizes the joint distribution in a way that reflects the hierarchical structure of the data-generative model, as given by (4).

### 3.3 | Model Assumptions

We pursue analytically accessible posterior distribution of all model parameters in (4) conditional on ϕ and $\delta^{2}$. The auxiliary model hyperparameters $\mu_{\beta },\;V_{\beta },\;\mu_{\gamma },\;V_{\gamma },\;a_{\tau },\;b_{\tau },\;a_{\sigma }$ and $b_{\sigma }$ are assumed fixed. Following (5), since β and $\tau^{2}$ are conditionally independent of all model components, given ($Y,Z_{L}$), we factorize the posterior distribution as

(7) $$\begin{array}{ll} p\left(Z_{L},Z_{\tilde{\ell }},\beta ,\gamma ,\tau^{2},\sigma^{2}|Y,X,\phi ,\delta^{2}\right)= & p\left(\beta ,\sigma^{2}|Z_{L},Y\right)\\ & \times \;p\left(Z_{L},Z_{\tilde{\ell }},\gamma ,\sigma^{2}|Y,X,\phi ,\delta^{2}\right) \end{array}$$

We make one additional assumption on $p\left(Z_{L},Z_{\tilde{\ell }},\gamma ,\sigma^{2}|Y\right.,\;X,\phi ,\delta^{2}$) as follows.

#### Assumption 1.

Under fixed values of ϕ and $\delta^{2}$, the latent spatial-temporal processes ($Z_{L},Z_{\tilde{𝒞}}$) is conditionally independent of the outcome Y, given noisy measurements of the covariate X, that is,

$$p\left(Z_{L},Z_{\tilde{\ell }},\gamma ,\sigma^{2}|Y,X,\phi ,\delta^{2}\right)=p\left(Z_{L},Z_{\tilde{\ell }},\gamma ,\sigma^{2}|X,\phi ,\delta^{2}\right).$$

**FIGURE 4 |:**
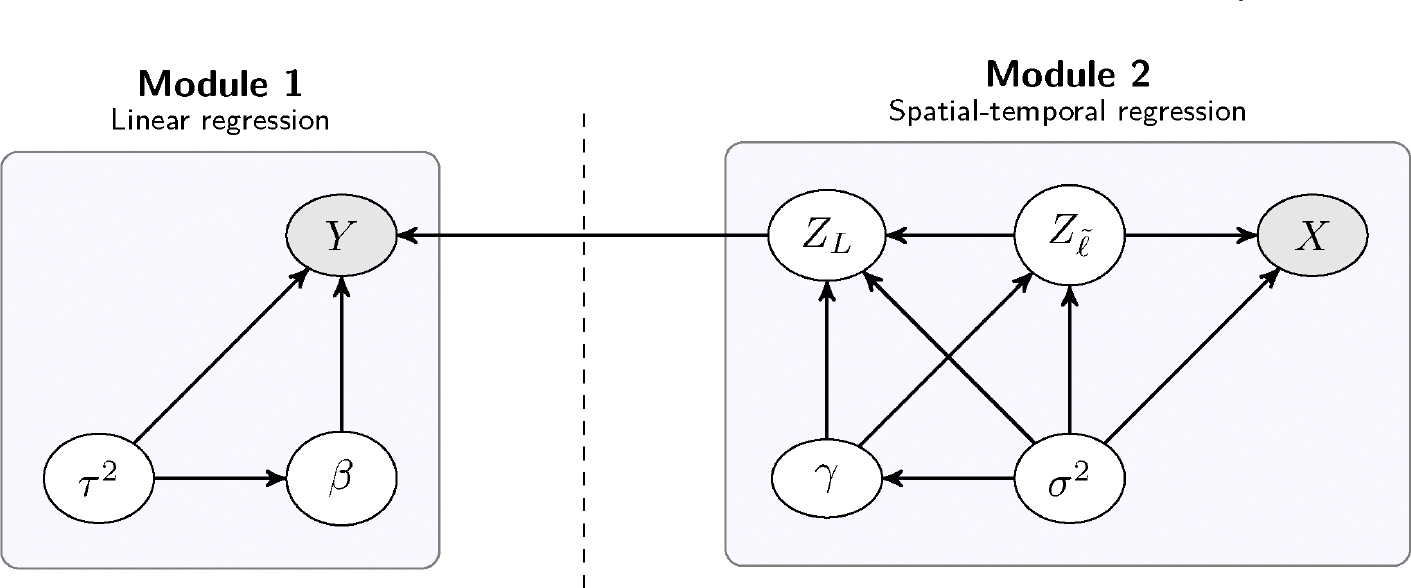
Directed acyclic graph (DAG) illustrating the conditional dependence structure of the hierarchical model (4). Nodes shaded in gray correspond to data that are observed; all other nodes represent unobserved (latent or unknown) quantities. The vertical dashed line denotes a “cut” in the DAG, indicating restricted flow of information from Y to Module 2 during posterior inference.

This implies that, instead of estimating a Bayesian full probability model, we assume that the latent spatial-temporal processes ($Z_{L},Z_{\tilde{\ell }}$) are a priori dependent, but once X is observed, Y provides no additional information about them. In the context of our analysis, Assumption 1 appears quite natural since it dictates that the estimation of annual county-level ozone concentrations only depends on X, that is, the monthly ozone levels measured by the monitoring stations, and does not depend on asthma ED visit rates Y. In the literature of Bayesian inference for complex hierarchical models, this is a familiar approach, especially when there are multiple data sources that provide information about different parameters in the model (see, e.g., Bayarri et al. 2009; Jacob et al. 2017). This is commonly known as *modularization*, as Assumption 1 modulates the flow of information from observed data to the latent process. Moreover, our proposed framework can also be viewed as a *cut model* (Plummer 2014), since it mimics a cut in the directed acyclic graph representing the hierarchical model, separating the graph into two components by logically preventing the “feedback” from one part of the model to the other during inference. We illustrate this in Figure 4.

The separate components of the model are often called *modules*. In this context, the hierarchical model (4) is made up of two modules – a linear regression module (Module 1), and a spatial-temporal regression module (Module 2). In a full probability model, the information from the outcome Y “feeds back” through the graph in Figure 4 to influence the posterior distribution of the latent spatial-temporal processes ($Z_{L},Z_{\tilde{\ell }}$). Instead, both $Z_{L}$ and $Z_{\tilde{\ell }}$ are estimated using the auxiliary data X. Cut models are also attractive from a computational viewpoint, as they significantly simplify sampling from the posterior distribution.

### 3.4 | Posterior Distribution

Following Assumption 1, conditional on ϕ and $\delta^{2}$, we derive that the posterior distribution corresponding to the hierarchical model in (4) as

(8) $$\begin{array}{l} N(\beta |M_{\beta }m_{\beta },\tau^{2}M_{\beta })\times IG(\tau^{2}|a_{\tau }^{*},b_{\tau }^{*})\times N(Z_{L}|\mu_{L|\tilde{\ell }},\sigma^{2}C_{L|\tilde{\ell }})\\ \;\times \;N(Z_{\tilde{\ell }}|M_{z}m_{z},\sigma^{2}M_{z})\times N(\gamma |M_{\gamma }m_{\gamma },\sigma^{2}M_{\gamma })\times IG(\sigma^{2}|a_{\sigma }^{*},b_{\sigma }^{*})\;, \end{array}$$

where $a_{\tau }^{*}=a_{\tau }+K/2$, $b_{\tau }^{*}=b_{\tau }+(Y^{⊤}D_{L}^{-1}Y+\mu_{\beta }^{⊤}V_{\beta }^{-1}\mu_{\beta }-m_{\beta }^{⊤}M_{\beta }m_{\beta })/2$, and $a_{\sigma }^{*}=a_{\sigma }+N/2,\;b_{\sigma }^{*}=b_{\sigma }+(X^{⊤}V_{X}^{-1}X+\mu_{\gamma }^{⊤}V_{\gamma }^{-1}\mu_{\gamma }-m_{\gamma }^{⊤}M_{\gamma }m_{\gamma })/2$, with $V_{X}=C_{\tilde{\ell }}(\phi )+\delta^{2}D_{\tilde{\ell }}$, and

(9) $$\begin{array}{l} M_{\beta }^{-1}={\tilde{W}}^{⊤}D_{L}^{-1}\tilde{W}+V_{\beta }^{-1},\;m_{\beta }={\tilde{W}}^{⊤}D_{L}^{-1}Y+V_{\beta }^{-1}\mu_{\beta }\;,\\ M_{\gamma }^{-1}={\tilde{\Psi }}^{⊤}V_{X}^{-1}\tilde{\Psi }+V_{\gamma }^{-1},\;m_{\gamma }={\tilde{\Psi }}^{⊤}V_{X}^{-1}X+V_{\gamma }^{-1}\mu_{\gamma }\;,\\ M_{z}^{-1}=C_{\tilde{\ell }}(\phi {)}^{-1}+(1/\delta^{2})D_{\tilde{\ell }}^{-1},\;m_{z}=(X-\tilde{\Psi }\gamma )/\delta^{2}\;, \end{array}$$

where $\tilde{W}=\left[W,Z_{L}\right]$ being the K×(p+1) matrix obtained by augmenting W by the vector $Z_{L}$ on the right. The first two terms in (8) denote the conditional posterior distributions $p\left(\beta |\tau^{2},Z_{L},Y\right)$ and $p\left(\tau^{2}|Z_{L},Y\right)$, respectively. The third term denote the posterior predictive distribution $p\left(Z_{L}|Z_{\tilde{\ell }},\gamma ,\sigma^{2},\phi ,\delta^{2}\right)$, with $\mu_{L|\tilde{\ell }}$ and $C_{L|\tilde{\ell }}$ as defined in (6). The fourth through sixth terms represent the conditional posterior distributions $p\left(Z_{\tilde{\ell }}|\gamma ,\sigma^{2},X,\phi ,\delta^{2}\right),p\left(\gamma |\sigma^{2},X,\phi ,\delta^{2}\right)$, and the marginal posterior distribution $p\left(\sigma^{2}|X,\phi ,\delta^{2}\right)$, respectively. This factorization follows from the fact that $Z_{L}$ is conditionally independent of the data X, given $Z_{\tilde{\ell }},\;\gamma ,\;\sigma^{2}$ and ϕ. It is important to remark that, the analytical tractability of the posterior distribution arises from the assumption that ϕ and $\delta^{2}$ are fixed and due to the condition specified in Assumption 1.

**ALGORITHM 1 |:** Posterior sampling.

1:	Compute $chol\left(V_{X}\right)$ given $\phi ,\;\delta^{2}$;	⊳ $𝒪(N^{3})$ flops
2:	Compute $chol\left(M_{z}\right)$ using $chol\left(V_{X}\right)$, and $M_{z}=\delta^{2}D_{\tilde{\ell }}V_{X}^{-1}C_{\tilde{\ell }}(\phi )$;	⊳ $𝒪(N^{3})$ flops
3:	Compute $chol(C_{\tilde{\ell }}(\phi ))$, then $chol(C_{L|\tilde{\ell }})$ using (6);	⊳ $𝒪(KN^{2}+K^{3})$ flops
4:	Compute $M_{\gamma },\;m_{\gamma }$, then $a_{\sigma }^{*},\;b_{\sigma }^{*}$;	⊳ $𝒪(rN^{2}+r^{3})$ flops
5:	**for** b=1 to $B_{\text{post}}$ **do**	
6:	Sample $\sigma^{2(b)}\sim IG(a_{\sigma }^{*},b_{\sigma }^{*})$	
7:	Sample $\gamma^{\left(b\right)}\sim N\left(M_{\gamma }m_{\gamma },\sigma^{2(b)}M_{\gamma }\right)$	
8:	Compute $m_{z}^{\left(b\right)}=(X-{\tilde{\Psi }}_{\gamma }^{\left(b\right)})/\delta^{2}$;	⊳ 𝒪(N) flops
9:	Sample $Z_{\tilde{\ell }}^{\left(b\right)}\sim N(M_{z}m_{z}^{\left(b\right)},\sigma^{2(b)}M_{z})$ using $chol\left(M_{z}\right)$;	⊳ $𝒪\left(N^{2}\right)$ flops
10:	Compute $\mu_{L|\tilde{\ell }}^{\left(b\right)}$ using $Z_{\tilde{\ell }}^{\left(b\right)}$ and $\gamma^{\left(b\right)}$ in (6);	⊳ $𝒪\left(KN^{2}\right)$ flops
11:	Sample $Z_{L}^{\left(b\right)}\sim N(\mu_{L|\tilde{\ell }}^{\left(b\right)},\sigma^{2(b)}C_{L|\tilde{\ell }})$;	⊳ 𝒪(N) flops
12:	Set ${\tilde{W}}^{\left(b\right)}=[W,Z_{L}^{\left(b\right)}]$, compute $M_{\beta }^{\left(b\right)},\;m_{\beta }^{\left(b\right)},\;a_{\tau }^{*(b)},\;b_{\tau }^{*(b)}$ using (9)	⊳ $𝒪\left(K^{3}\right)$ flops
13:	Sample $\tau^{2(b)}\sim IG(a_{\tau }^{*(b)},b_{\tau }^{*(b)})$	
14:	Sample $\beta^{\left(b\right)}\sim N(M_{\beta }^{\left(b\right)}m_{\beta }^{\left(b\right)},\tau^{2(b)}M_{\beta }^{\left(b\right)})$	
15:	**end for**	
16:	**return** $B_{\text{post}}$ samples ${\left\{\sigma^{2(b)},\gamma^{\left(b\right)},Z_{\tilde{\ell }}^{\left(b\right)},Z_{L}^{\left(b\right)},\tau^{2(b)},\beta^{\left(b\right)}\right\}}_{b=1}^{B}$ from the posterior distribution (8).	

We use composition sampling to draw samples from the posterior distribution in (8). We elaborate the steps in Algorithm 1. Here, chol(⋅) refers to the lower-triangular Cholesky factor of a square matrix.

The steps in Algorithm 1, generate $B_{\text{post}}$ samples ${\left\{\sigma^{2(b)},\gamma^{\left(b\right)},Z_{\tilde{\ell }}^{\left(b\right)},Z_{L}^{\left(b\right)},\tau^{2(b)},\beta^{\left(b\right)}\right\}}_{b=1}^{B_{\text{post}}}$ from the posterior distribution. The sampling algorithm is dominated by Cholesky decompositions of N×N matrices $V_{X},\;M_{z}$, and $C_{\tilde{\ell }}(\phi )$, which accumulates $𝒪\left(N^{3}\right)$ floating-point operations (flops). The matrix $M_{z}$ is calculated efficiently using the identity $M_{z}=\delta^{2}D_{\tilde{\ell }}V_{X}^{-1}C_{\tilde{\ell }}(\phi )$, where $V_{X}^{-1}C_{\tilde{\ell }}(\phi )$ is calculated using triangular solves of columns of $C_{\tilde{\ell }}(\phi )$ with respect to the Cholesky factor already calculated $chol\left(V_{X}\right)$. Placing a prior on ϕ and the nugget $\theta =\delta^{2}\sigma^{2}$ requires iterative algorithms such as MCMC to sample from the posterior distribution, which entails repeated Cholesky decompositions of these N×N matrices. Moreover, weak identifiability of $\phi ,\;\sigma^{2}$ and θ impedes convergence of the random walk Metropolis steps, which contributes to delayed execution times. Thus, even for moderately sized datasets $\left(N\sim 10^{3}\right)$, the computation becomes too onerous for practical use and alternative strategies like low-rank models are used (Finley et al. 2015). In this context, predictive stacking presents itself as an effective alternative by enhancing the practicality of full Gaussian process models for moderately large datasets.

#### Remark 1.

Given observations at $\tilde{ℒ}$, let $ℒ=\{\ell_{i}=\left(s_{i},t_{i}\right):i=1,\;\ldots ,N^{*}\}$ be a collection of $N^{*}$ space-time coordinates in 𝒟, where we wish to predict latent spatial-temporal process. Define $N^{*}\times 1$ vector $Z_{\ell }={\left(Z\left(\ell_{1}\right),\;\ldots ,Z\left(\ell_{N^{*}}\right)\right)}^{⊤}$. Then, posterior predictive inference at ℒ follows from

(10) $$p\left(Z_{\ell }|X,\phi ,\delta^{2}\right)=\int p\left(Z_{\ell }|Z_{\tilde{\ell }},\gamma ,\sigma^{2},\phi ,\delta^{2}\right)\;p\left(Z_{\tilde{\ell }},\gamma ,\sigma^{2}|X,\phi ,\delta^{2}\right)\;dZ_{\tilde{\ell }}\;d\gamma \;d\sigma^{2}\;,$$

where the conditional density $p(Z_{\ell }|Z_{\tilde{\ell }},\gamma ,\sigma^{2},\phi ,\delta^{2})=N(Z_{\ell }|\mu_{\ell |\tilde{\ell }},\sigma^{2}C_{\ell |\tilde{\ell }})$, with

(11) $$\begin{array}{l} \mu_{\ell |\tilde{\ell }}=\Psi_{\gamma }+C_{\ell ,\tilde{\ell }}\left(\phi \right)C_{\tilde{\ell }}^{-1}\left(\phi \right)\left(Z_{\tilde{\ell }}-{\tilde{\Psi }}_{\gamma }\right)\;,\\ C_{\ell |\tilde{\ell }}=C_{\ell }(\phi )-C_{\ell ,\tilde{\ell }}(\phi )C_{\tilde{\ell }}(\phi {)}^{-1}C_{\ell ,\tilde{\ell }}(\phi {)}^{⊤}\;, \end{array}$$

where ${\left(\mu_{\ell }(\gamma )\right)}_{i}=\mu \left(\ell_{i};\gamma \right)$, and $N^{*}\times N$ matrix $C_{\ell ,\tilde{\ell }}(\phi )$, and $N^{*}\times N^{*}$ matrix $C_{\ell }(\phi )$ are defined as

$${\left(C_{\ell }(\phi )\right)}_{i,i^{\prime }}\;=C\left(\ell_{i},\ell_{i^{\prime }};\phi \right),\;{\left(C_{\ell ,\tilde{\ell }}(\phi )\right)}_{i,j}\;={\left|{\tilde{I}}_{j}\right|}^{-1}\int_{{\tilde{I}}_{j}}C\left(\left(s_{i},t_{i}\right),\left({\tilde{s}}_{j},t\right);\phi \right)dt\;,$$

for $i,i^{\prime }=1,\;\ldots ,N^{*}$ and j=1,…,N. We sample from (10) by first drawing $\left\{Z_{\tilde{\ell }}^{\left(b\right)},\gamma^{\left(b\right)},\sigma^{2(b)}\right\}$ from $p(Z_{\tilde{\ell }},\gamma ,\sigma^{2}|X,\phi ,\delta^{2})$ using Algorithm 1. Then, for each drawn value of $\left\{Z_{\tilde{\ell }}^{\left(b\right)},\gamma^{\left(b\right)},\sigma^{2(b)}\right\}$, we sample $Z_{\ell }$ from $N(Z_{\ell }|\mu_{\ell |\tilde{\ell }}^{\left(b\right)},\sigma^{2(b)}C_{\ell |\tilde{\ell }})$, where $\mu_{\ell |\tilde{\ell }}^{\left(b\right)}$ is obtained by substituting $Z_{\tilde{\ell }}$ and γ by $Z_{\tilde{\ell }}^{\left(b\right)}$ and $\gamma^{\left(b\right)}$, respectively, in (11). Repeating this for b=1,…,B yields samples $\left\{Z_{\ell }^{\left(b\right)}:b=1,\;\ldots ,B\right\}$ from (10).

### 3.5 | Spatial-Temporal Correlation Function

As seen from (3), evaluation of the elements of $C_{\tilde{\ell }}(\phi )$ involves numerical integrations over the temporal domain. Popular methods for univariate numerical integration (e.g., trapezoidal/Simpson’s rule, quadrature, Monte Carlo) for a smooth function incur 𝒪(M) operations, where M is the number of knots or evaluation points. Thus, computing all elements of $C_{\tilde{\ell }}(\phi )$ would require $𝒪\left(MN^{2}\right)$ flops, which can be expensive for moderately large N. Therefore, we explore spatial-temporal correlation functions that offer improved tractability, especially over the temporal domain.

In particular, we assume a *separable* spatial-temporal correlation function (Mardia and Goodall 1993) of the form $C\left(\ell ,\ell^{\prime };\phi \right)=C_{s}\left(s,s^{\prime };\phi_{1},v\right)\cdot C_{t}\left(t,t^{\prime };\phi_{2}\right)$, where $C_{s}\left(s,s^{\prime };\phi_{1},v\right)$ and $C_{t}\left(t,t^{\prime };\phi_{2}\right)$ denote the isotropic Matérn and the exponential correlation functions, respectively, given by

(12) $$\begin{array}{l} C_{s}\left(s,s^{\prime };\phi_{1},v\right)=\frac{{\left(\phi_{1}\left\|s\;-\;s^{\prime }\right\|\right)}^{v}}{2^{v-1}\Gamma (v)}K_{v}\left(\phi_{1}\left\|s-s^{\prime }\right\|\right),\\ \;C_{t}\left(t,t^{\prime };\phi_{2}\right)=\exp\left(-\phi_{2}\left|t-t^{\prime }\right|\right), \end{array}$$

where $\left\|s-s^{\prime }\right\|$ is the Euclidean distance between $s,\;s^{\prime }\in 𝒮$. The function Γ(⋅) denotes the gamma function, and $K_{v}$ is the modified Bessel function of the second kind of order v which may be fractional (Abramowitz and Stegun 1965, chapter 10). Hence, in this case, $\phi =\left(\phi_{1},v,\phi_{2}\right)$ is a 3-dimensional. The assumption of *separability* conveniently splits space and time in calculation of covariance matrices $C_{\tilde{\ell }}(\phi ),C_{L}(\phi )$, and the cross-covariance matrix $C_{\tilde{\ell },L}(\phi )$. For example, the elements of $C_{\tilde{\ell }}(\phi )$ are

(13) $${\left(C_{\tilde{\ell }}(\phi )\right)}_{j,j^{\prime }}={\left(\left|{\tilde{I}}_{j}\right|\left|{\tilde{I}}_{j^{\prime }}\right|\right)}^{-1}C_{s}\left({\tilde{s}}_{j},{\tilde{s}}_{j^{\prime }};\phi_{1},v\right)\int_{{\tilde{I}}_{j^{\prime }}}\int_{{\tilde{I}}_{j}}C_{t}\left(t,t^{\prime };\phi_{2}\right)dtdt^{\prime }.$$

We make an additional assumption that facilitates closed form expression for the integral in (13). The distribution theory in Section 3.1 applies to any Borel-measurable subsets ${\tilde{I}}_{j}$ and $I_{k}$. In practice, however, it is natural to assume that observations are aggregated over a single interval.

#### Assumption 2.

The temporal blocks ${\tilde{I}}_{j}$ for j=1,…,N and $I_{k}$ for k=1,…,K, are of the form ${\tilde{I}}_{j}=\left({\tilde{a}}_{j},{\tilde{b}}_{j}\right)$, and $I_{k}=\left(a_{k},b_{k}\right)$ where ${\tilde{a}}_{j}<{\tilde{b}}_{j}$ for each j and $a_{k}<b_{k}$ for each k.

#### Proposition 1.

*For any* a<b *and* c<d, *suppose* ${\tilde{C}}_{t}(a,b,c,d;\phi )=\int_{c}^{d}\int_{a}^{b}C_{t}\left(t,t^{\prime };\phi_{2}\right)dtdt^{\prime }$, *then*

- *for non-overlapping* (a,b) *and* (c,d), *with* a<b≤c<d,
$${\tilde{C}}_{t}\left(a,b,c,d;\phi_{2}\right)=\frac{1}{\phi_{2}^{2}}\left[F\left(a,d\right)+F\left(b,c\right)-F\left(a,c\right)-F\left(b,d\right)\right]\;,$$
- *if intervals* (a,b) *and* (c,d) *overlap, with* a≤c<b≤d,
$$\begin{array}{ll} {\tilde{C}}_{t}\left(a,b,c,d;\phi_{2}\right)= & \frac{1}{\phi_{2}^{2}}\left[2\phi_{2}(b-c)+F(a,d)+F(c,b)\right.\\ & \;-\;F(a,c)-F(b,d)]\;, \end{array}$$
- *if* (a,b) *is nested within* (c,d), *that is, either* c≤a<b<d *or* c<a<b≤d,
$$\begin{array}{ll} {\tilde{C}}_{t}\left(a,b,c,d;\phi_{2}\right)= & \frac{1}{\phi_{2}^{2}}\left[2\phi_{2}(b-a)+F(a,d)+F(c,b)\right.\\ & \;-\;F(c,a)-F(b,d)]\;, \end{array}$$
  *where* $F\left(a_{1},a_{2}\right)=F\left(a_{1},a_{2};\phi_{2}\right)=exp\left(-\phi_{2}\left(a_{2}-a_{1}\right)\right)$ *for any* $a_{1},\;a_{2}\in 𝒯$.

##### Proof.

See Section S3 of the Supporting Information for details. □

We benefit from the customary Assumption 2 that the integral of $C_{t}\left(\cdot ,\cdot ;\phi_{2}\right)$ as appearing in (13) admits closed-form expressions, as detailed in Proposition 1. This facilitates a rapid evaluation of elements of the spatial-temporal covariance matrix $C_{\tilde{\ell }}(\phi )$ without resorting to approximations using numerical methods. However, Proposition 1 does not extend to similar results for integrations over irregular spatial blocks (e.g., counties of California), and hence numerical methods are essential for computing elements of $C_{L}(\phi )$ and $C_{\tilde{\ell },L}(\phi )$. In this context, assuming the separable spatial-temporal covariance function (12) provides additional advantage, as we are able to write the integral as a product of a spatial and a temporal component. Following (3) and Assumption 2, we have

(14) $$\begin{array}{l} {\left(C_{\tilde{\ell },L}(\phi )\right)}_{j,k}=\frac{{\left|B_{k}\right|}^{-1}}{({\tilde{b}}_{j}\;-\;{\tilde{a}}_{j})\left(b_{k}\;-\;a_{k}\right)}{\tilde{C}}_{t}\left({\tilde{a}}_{j},{\tilde{b}}_{j},a_{k},b_{k}\right)\int_{B_{k}}C_{s}\left({\tilde{s}}_{j},s\right)ds\;,\\ {\left(C_{L}(\phi )\right)}_{k,k^{\prime }}=\frac{{\left(\left|B_{k}||B_{k^{\prime }}\right|\right)}^{-1}}{\left(b_{k}\;-\;a_{k}\right)\left(b_{k^{\prime }}\;-\;a_{k^{\prime }}\right)}{\tilde{C}}_{t}\left(a_{k},b_{k},a_{k^{\prime }},b_{k^{\prime }}\right)\int_{B_{k^{\prime }}}\int_{B_{k}}C_{s}\left(s,s^{\prime }\right)dsds^{\prime }\;, \end{array}$$

for each j=1,…,N and $k,\;k^{\prime }=1,\;\ldots ,K$. The role of Proposition 1 becomes clear from (14), as it helps to simplify (3) by reducing the necessity of any numerical integration in the time domain. From a computational perspective, Proposition 1 implies that evaluating $C_{\tilde{\ell }}(\phi )$ requires no numerical integration, $C_{\tilde{\ell },L}(\phi )$ requires NK integrals, and $C_{L}(\phi )$ requires $K^{2}$ integrals. In practice, K is much smaller than N; for example, California has only 58 counties, and we include data for 5 racial groups over 8 years (2015–2022), yielding K=1510 after removing missing records. In contrast, ozone levels are recorded at N=15,725 spatial-temporal coordinates.

## 4 | Predictive Stacking

### 4.1 | Choice of Candidate Models

To implement stacking, we first fix the values of the hyperparameters of the auxiliary model. In practice, we assume $\mu_{\beta }=0_{p+1},\;V_{\beta }=\delta_{\beta }^{2}I_{p+1}$ for a sufficiently large $\delta_{\beta }$ to specify a weakly informative prior for the fixed effects β. Here, $0_{p+1}$ denotes the zero vector of length p+1. Similarly, we assume $\mu_{\gamma }=0_{r}$ and $V_{\gamma }=\delta_{\gamma }^{2}I_{r}$ for the Gaussian prior on γ. For $\tau^{2}$ and $\sigma^{2}$, we choose the shape parameters $a_{\tau }=a_{\sigma }=2$ and the scale parameters $b_{\tau }=b_{\sigma }=0.1$.

For $\phi =\left(\phi_{1},\phi_{2},v\right)$ and $\delta^{2}$, we choose grids of candidate values given by $G_{\phi_{1}},\;G_{\phi_{2}},\;G_{\nu }$ and $G_{\delta^{2}}$. The grid $G_{\delta^{2}}$ is chosen based on the values of the nugget and partial sill, estimated from an empirical semivariogram. L. Zhang et al. (2025) provides further details on how the quantiles of a Beta distribution specified by the estimated values of the nugget and the partial sill can be used to provide useful information for selecting $G_{\delta^{2}}$. On the other hand, the candidate values of $\phi_{1}$ and $\phi_{2}$ are chosen so that the “effective range” (distance at which the correlation drops below 5%) corresponding to the candidate values is between 20% and 70% ofthe maximum distance between the space-time coordinates (see, chapter 2, Banerjee et al. 2025). We choose $G_{v}$ to include some customary values of the Matérn smoothness parameter that are common in spatial analysis. For example, $G_{v}=\{0.5,\;1.0,\;1.5\}$.

### 4.2 | Stacking Algorithm

While we follow the general strategy of stacking predictive densities as proposed in Yao et al. (2018), our development is distinct in that we adapt it to our more complex model, which differs significantly from the standard spatial-temporal modeling frameworks considered in the previous literature (Zhang et al. 2025; Pan et al. 2026). In this aspect, Assumption 1 plays a key role, as it restricts the inference for the latent spatial-temporal process to Module 2, within which we seek to find an optimal way to combine the inference conditional on candidate values of ϕ and $\delta^{2}$. We elaborate below.

Let $ℳ=\left\{M_{1},\;\ldots ,M_{G}\right\}$ denote a collection of candidate models G, where $M_{g}$ corresponds to fixed values of the parameters $\left(\phi_{g},\delta_{g}^{2}\right)$, for g=1,…,G. Predictive stacking finds a probability distribution $\tilde{p}$ in the class $𝒞=\{\sum_{g=1}^{G}\alpha_{g}p\left(\cdot |X,M_{g}\right):\sum_{g=1}^{G}\alpha_{g}=1,\alpha_{g}\geq 0\}$, such that the Kullback–Leibler (KL) divergence between $\tilde{p}(\cdot |X)$ and $p_{t}(\cdot |X)$ is minimized, where $p_{t}$ denotes the posterior predictive distribution under the true data-generating model. Here, $p\left(\cdot |X,M_{g}\right)$ denotes the posterior predictive distribution under the candidate model $M_{g}$, for each g. We define the stacking weights $\alpha =\left(\alpha_{1},\;\ldots ,\alpha_{G}\right)$ as the solution to the optimization problem

(15) $$\begin{array}{l} \underset{\alpha }{max}\frac{1}{N}\sum_{j=1}^{N}log\sum_{g=1}^{G}\alpha_{g}p\left(X\left({\tilde{\ell }}_{j}\right)|X_{-j},M_{g}\right)\\ \;\text{subject}\;\text{to}\;\alpha^{⊤}1_{G}=1,\alpha \in {\left[0,\;1\right]}^{G}, \end{array}$$

where $X_{-j}$ denotes the data X with the j th observation removed, and $p\left(X\left({\tilde{\ell }}_{j}\right)|X_{-j},M_{g}\right)$ denotes the leave-one-out predictive density corresponding to the j th observation. This follows from a result that establishes that minimizing $KL\left(\tilde{p}(\cdot |X),p_{t}(\cdot |X)\right)$ under the constraint $\tilde{p}\in 𝒞$ is asymptotically equivalent to the optimization problem in (15) (see, Le and Clarke 2017; Clyde and Iversen 2013). The optimization task in (15) falls into the class of convex problems and can be formulated and solved using suitable modeling tools and solvers. Assumption 1 implies that the optimal stacking weights can be computed solely based on Module 2, that is, using only X. Posterior inference for quantities of interest subsequently proceeds from the “stacked posterior”,

(16) $$\tilde{p}(\cdot |X,Y)=\sum_{g=1}^{G}{\overset{ˆ}{\alpha }}_{g}p\left(\cdot |X,Y,M_{g}\right),$$

where ${\overset{ˆ}{w}}_{g}$ denotes optimal stacking weights obtained by solving the optimization task in (15).

An important prerequisite for evaluating the objective function in (15) is finding the leave-one-out predictive densities. Under the model (4), the leave-one-out predictive densities admit a closed form,

(17) $$\begin{array}{l} \;p\left(X\left({\tilde{\ell }}_{j}\right)|X_{-j},M_{g}\right)\\ =t_{2a_{\sigma ,j}^{*}}\left(X\left({\tilde{\ell }}_{j}\right)|\mu_{g,j|-j}\left({\tilde{\ell }}_{j}\right),\left(b_{\sigma ,j}^{*}/a_{\sigma ,j}^{*}\right)\sigma_{g,j|-j}\right), \end{array}$$

where $t_{\rho }\left(x;m,v^{2}\right)$ denotes the location-scale t-density with degrees of freedom ρ, location m and scale v, evaluated at x, for each j. The parameters $\mu_{g,j|-j}\left({\tilde{\ell }}_{j}\right)$ and $\sigma_{g,j|-j}$ are given by

$$\begin{array}{ll} \mu_{g,j|-j}\left({\tilde{\ell }}_{j}\right) & =R_{g,j}^{⊤}V_{g,X_{-j}}X_{-j}+H_{g,j}^{⊤}M_{\gamma ,g,j}m_{\gamma ,g,j}\;,\\ \sigma_{g,j|-j} & =V_{g,X_{j}|X_{-j}}+H_{g,j}^{⊤}M_{\gamma ,g,j}H_{g,j}\;, \end{array}$$

where $R_{g,j}$ is the (N-1)×1 spatial-temporal cross-correlation matrix between $\tilde{ℒ}\backslash {\tilde{\ell }}_{j}$ and $\left\{{\tilde{\ell }}_{j}\right\}$ under $M_{g}$, and is given by the j th column of $C_{\tilde{\ell },-j}\left(\phi_{g}\right)$ which denotes the matrix $C_{\tilde{\ell }}\left(\phi_{g}\right)$ with both its j th row and column removed. Moreover, the matrix $V_{g,X_{-j}}$ is obtained by removing the j th row and column of $V_{g,X}=C_{\tilde{\ell }}\left(\phi_{g}\right)+\delta_{g}^{2}D_{\tilde{\ell }}$. In addition, $M_{\gamma ,g,j}^{-1}={\tilde{\Psi }}_{-j}^{⊤}V_{g,X_{-j}}{\tilde{\Psi }}_{-j}+V_{\gamma }^{-1}$, and $m_{\gamma ,g,j}={\tilde{\Psi }}_{-j}V_{g,X_{-j}}X_{-j}+V_{\gamma }^{-1}\mu_{\gamma }$ where the (N-1)×r matrix ${\tilde{\Psi }}_{-j}$ is obtained by deleting the j th row of $\tilde{\Psi }$. The r×1 vector $H_{g,j}=\tilde{\psi }\left({\tilde{\ell }}_{j}\right)-{\tilde{\Psi }}_{-j}^{⊤}V_{g,X_{-j}}^{-1}R_{g,j}$, and the scalar $V_{g,X_{j}|X_{-j}}={\left(V_{g,X}\right)}_{j,j}-R_{g,j}^{⊤}V_{g,X_{-j}}R_{g,j}$ with ${\left(V_{g,X}\right)}_{j,j}$ denoting the j th diagonal element of $V_{g,X}$. Furthermore, $a_{\sigma ,g}^{*}=a_{\sigma }+(N-1)/2$, and $b_{\sigma ,g}^{*}=b_{\sigma }+(X_{-j}^{⊤}V_{g,X_{-j}}X_{-j}+\mu_{\gamma }^{⊤}V_{\gamma }^{-1}\mu_{\gamma }-m_{\gamma ,g,j}^{⊤}M_{\gamma ,g,j}m_{\gamma ,g,j})/2$.

Evaluation of (17) is dominated by the Cholesky decomposition of the (N-1)×(N-1) matrix $V_{g,X_{-j}}$, which requires $𝒪\left(N^{3}\right)$, for each j. Hence, a naive approach to find the leave-one-out predictive densities under each $M_{g}$ results in $\sim 𝒪\left(N^{4}\right)$ flops, which is impractical. We mitigate this issue by reusing the Cholesky factor of $V_{g,X}$ which has already been computed once while fitting the model $M_{g}$ (Kim et al. 2002). Subsequently, we compute the Cholesky factor of $V_{g,X_{-j}}$ for each j using an efficient rank-one update algorithm (Krause and Igel 2015), which ultimately accumulates to $𝒪\left(N^{3}\right)$ flops, thereby delivering a significant speedup over the naive approach.

Although rank-one updates are faster than the naive method, they are computationally expensive; hence, an alternative approach is desirable. Here, importance weighting is an attractive option to approximate leave-one-out predictive densities (see, e.g., Gelfand et al. 1992; Vehtari, Simpson, et al. 2024). Suppose ${\left\{Z_{\tilde{\ell },g}^{\left(b\right)},\sigma_{g}^{2(b)}\right\}}_{b=1}^{B_{\text{post}}}$ denotes $B_{\text{post}}$ draws from $p\left(Z_{\tilde{\ell }},\sigma^{2}|X,M_{g}\right)$, then for each j, we approximate the leave-one-out predictive densities by the weighted mean

(18) $$p\left(X\left({\tilde{\ell }}_{j}\right)|X_{-j},M_{g}\right)\approx \frac{1}{\sum_{b=1}^{B_{\text{post}}}r_{j,g}^{b}}\sum_{b=1}^{B_{\text{post}}}r_{j,g}^{b}\;N\left(X\left({\tilde{\ell }}_{j}\right)|Z_{\tilde{\ell },g,j}^{\left(b\right)},\sigma_{g}^{2(b)}\right),$$

where $Z_{\tilde{\ell },g,j}^{\left(b\right)}$ denotes the j th element of $Z_{\tilde{\ell },g}^{\left(b\right)}$, and $r_{j,g}^{b}$ is the important ratio defined as $1/r_{j,g}^{b}=N(X\left({\tilde{\ell }}_{j}\right)|Z_{\tilde{\ell },g,j}^{\left(b\right)},\sigma_{g}^{2(b)})$. The weights $r_{j,g}^{b}$ tend to have a high or infinite variance, introducing instability in the computation (18). To address these issues, Vehtari et al. (2017) proposes stabilizing the weights by fitting a generalized Pareto distribution to the tail of the weight distribution using the empirical Bayes estimation algorithm proposed in J. Zhang and Stephens (2009). Thus, no additional model fitting is necessary to calculate leave-one-out predictive densities. Compared to $𝒪\left(N^{3}\right)$ for the Cholesky factor update algorithm, the computational cost of this approximate method is O(NlogN) and therefore is much faster. Figure S5 of the Supporting Information compares exact and approximate leave-one-out predictive densities computed using the closed-form expression (17) and Pareto smoothed importance sampling, respectively. Both methods deliver practically indistinguishable results but differ in computational cost; exact computation requires roughly 3 min, whereas importance sampling needs only 0.1 s. We implement this using the R package loo (Vehtari, Gabry, et al. 2024).

## 5 | Simulation

### 5.1 | Simulated Data

**FIGURE 5 |:**
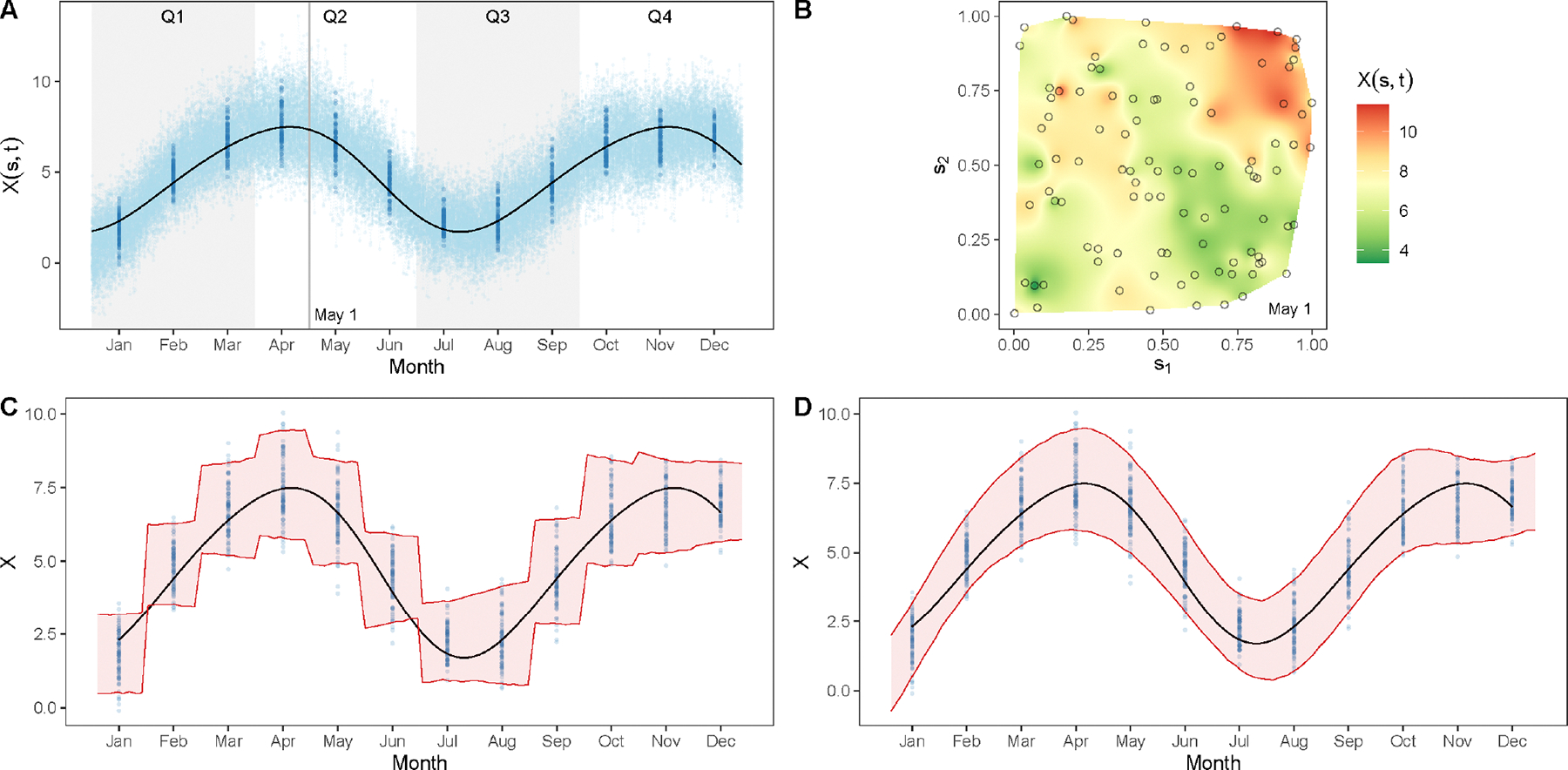
A: Light blue dots represents simulated daily measurements at 100 locations over a year, whereas dark blue dots denote monthly averages at each site. B: Interpolated spatial surface of a snapshot of the data (denoted by the vertical line in subfigure A) at May 1. C, D: 95% credible interval of posterior predictive samples drawn from the stacked posterior $\tilde{p}(Z(\ell )|X)$ with ψ(⋅) chosen as monthly factors and Fourier series as basis, respectively, to capture seasonal variations.

To address the analytical challenges posed by data that are simultaneously spatially and temporally misaligned, we designed a simulation study that closely replicates the structure and characteristics of the outcome (asthma) and the exposure (ozone) data, as discussed in Section 2. For the exposure data, we consider the unit square, that is, [0, 1]^2^, and the interval [0, 12] as the spatial and temporal domains of interest. The interval [0, 12] represents the duration of twelve months of a year. We simulate spatially-temporally correlated data at $n_{s}=100$ locations sampled uniformly in [0, 1]^2^, and at $n_{t}=\;360$ equally spaced time points in [0, 12], which amounts to spatial-temporal coordinates $\left\{\ell_{1},\;\ldots ,\ell_{n_{s}n_{t}}\right\}$ with $n_{s}n_{t}=36,\;000$. The data is simulated following the model $X\left(\ell_{i}\right)=Z\left(\ell_{i}\right)+e_{i}^{\prime }$ for $i=1,\;\ldots ,n_{s}n_{t}$, where $e_{i}^{\prime }\overset{\text{ind}}{\sim }N(0,\;1)$ denotes the measurement error for each i and $Z(\ell )\sim GP\left(\mu (\ell ),C\left(\ell ,\ell^{\prime };\phi \right)\right)$. The covariance function $C\left(\ell ,\ell^{\prime };\phi \right)$ is the same as (12) with $\phi_{1}=4,\;v=$ 0.5 and $\phi_{2}=0.6$. We choose the mean function μ(ℓ) to depend only on time and therefore write it as μ(t). We model μ(t) as a random draw from $GP\left(5,4\cdot R\left(t,t^{\prime };p,\lambda \right)\right)$, where $R\left(t,t^{\prime };p,\lambda \right)=exp\left(-2\lambda^{2}{sin}^{2}\left(\pi \left|t-t^{\prime }\right|/p\right)\right)$ denotes a periodic covariance kernel with period p=7 and decay λ=0.1. This helps introduce seasonal variations in the simulated data. Thus, the simulated data $X\left(\ell_{i}\right)$ for $i=1,\;\ldots ,n_{s}n_{t}$ mimic daily measurements of a variable at 100 monitoring sites over a year. From these, we compute monthly averages at each site, which we denote by $X\left({\tilde{\ell }}_{j}\right)$ for $j=1,\;\ldots ,12n_{s}$. To introduce missingness, we randomly remove 10% of the data from the monthly averages and denote the remaining data simply by X. We proceed to our simulation experiment with these point-referenced monthly averages as the data. See Figure 5A,B for a visualization of the simulated data.

For subsequent analysis, we consider the grids of candidate values of the spatial decay parameter $G_{\phi_{1}}=\{2,\;3,\;5\}$, $G_{\phi_{2}}=\{0.3,\;0.5,\;1\}$, $G_{v}=\{0.5,\;1,\;1.5\}$, and $G_{\delta^{2}}=\{0.75,\;1.5\}$. Hence, we stack on 3 × 3 × 3 × 2 = 54 models.

In addition to simulating the point-level latent process following the procedure in Section 5.1, we also generate the corresponding block-level quarterly latent process for each spatial unit. Specifically, for each block $L_{k}$, we simulate $X\left(L_{k}\right)$ using the joint Gaussian process model in (2), where the integrated block-level covariance terms are approximated via Monte Carlo averaging based on 500 uniformly drawn within-polygon samples. Subsequently, we generate the outcome $Y\left(L_{k}\right)=w{\left(L_{k}\right)}^{⊤}\beta_{1}+\beta_{2}X\left(L_{k}\right)+g\left(L_{k}\right)+\epsilon_{k}$, where $w\left(L_{k}\right)$ comprises an intercept and a predictor drawn independently from a standard normal distribution. The field $g\left(L_{k}\right)$ is drawn from the same covariance kernel as $X\left(L_{k}\right)$ and constructed to have varying correlation ρ∈{0.1,0.5,0.9} with $X\left(L_{k}\right)$. This term is included to model unmeasured spatial-temporal confounding.

### 5.2 | Point-Level Spatial-Temporal Prediction

As a first step, we examine the stacked posterior predictive distribution $\tilde{p}(X(\ell )|X)$, which corresponds to predicting the underlying process at a finer temporal resolution (e.g., daily) from coarser, aggregated observations (see Remark 1). In this context, ψ(⋅) in the mean function ψ(⋅) of the latent spatial-temporal process plays a key role. Under the assumption that $\mu (\ell )=\psi (t{)}^{⊤}\gamma$ depends only on time, we study the two alternative specifications of the mean function

(19) $$\begin{array}{ll} \mu^{\left(1\right)}(t) & =\sum_{v=1}^{r}\psi_{v}^{\left(1\right)}(t)\gamma_{v}\\ & =\gamma_{1}+\sum_{v=2}^{r}\gamma_{v}I\left(t\in {month}_{v}\right),\\ \mu^{\left(2\right)}(t) & =\sum_{v=1}^{r}\psi_{v}^{\left(2\right)}(t)\gamma_{v}\\ & =\gamma_{1}+\sum_{v=1}^{\left\lfloor r/2\right\rfloor }\gamma_{2v}\;sin\left(2\pi t/p_{v}\right)+\gamma_{2v+1}\;cos\left(2\pi t/p_{v}\right), \end{array}$$

where I(⋅) denotes the indicator function, ${month}_{v}$ denotes the interval (v-1,v) for v=2,…,12. For example, v=2 corresponds to the month February. In this case, r=12 and $\psi^{\left(1\right)}(t)$ corresponds to a simple monthly indicator basis function, which corresponds to monthly factors under monthly aggregated data.

On the other hand, $\mu^{\left(2\right)}(t)$ consists of smooth periodic basis functions, such as sine and cosine terms with different periodicity. For our analysis, we choose r=9 with $p_{v}=3+v$ for v=1,…,4. Figure 5C,D illustrates the behavior of the posterior predictive distribution under the two choices of ψ(⋅). We notice that the smooth basis functions lead to superior reconstruction of the latent process compared to discontinuous monthly indicator functions, due to their ability to borrow strength across adjacent time points and capture underlying seasonal trends more effectively.

### 5.3 | Block-Level Spatial-Temporal Prediction

**FIGURE 6 |:**
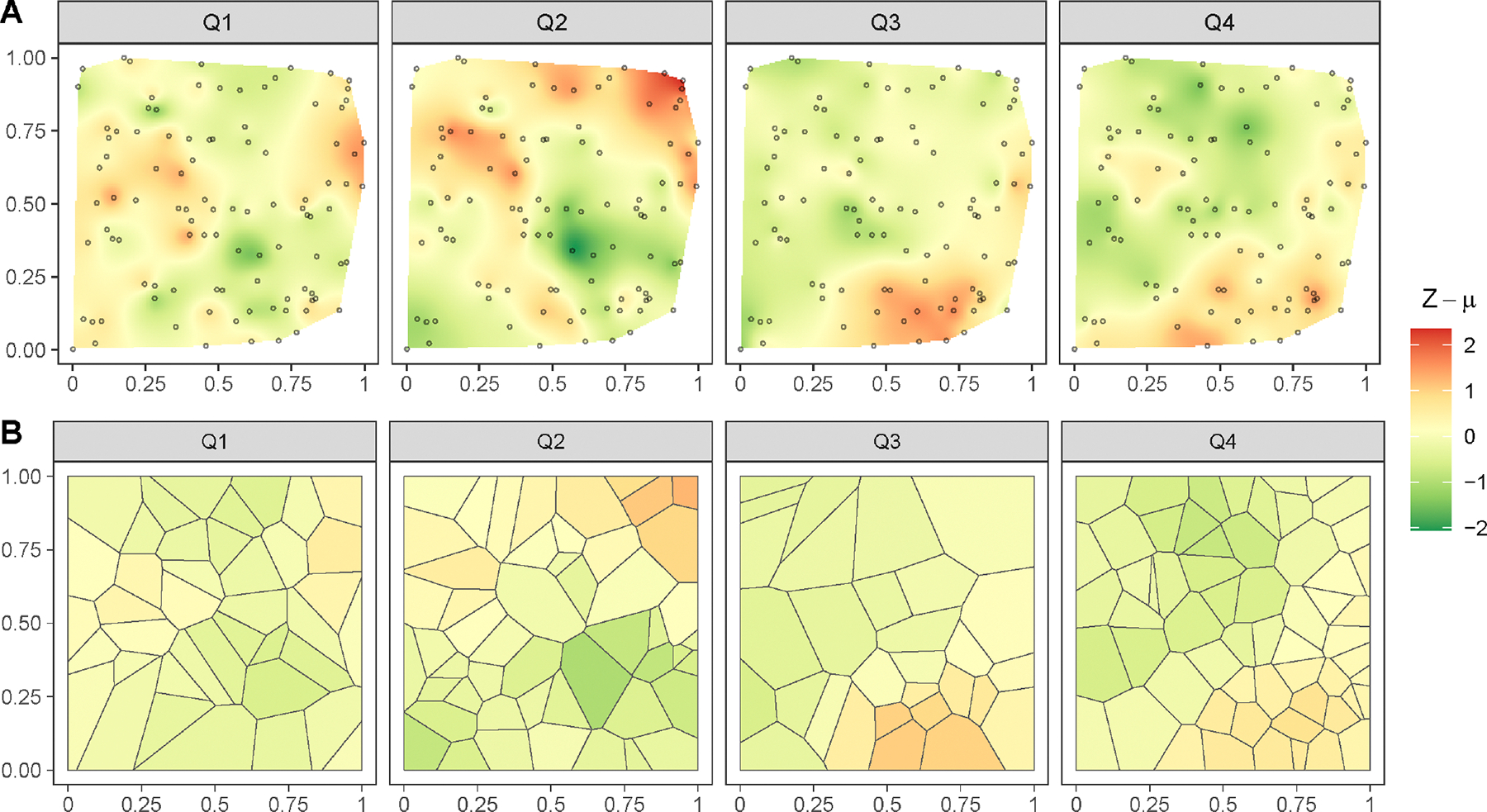
A: Interpolated spatial surfaces of the quarterly averaged de-trended true spatial-temporal process which simulated the data; B: Median of the stacked posterior predictive distribution $\left[\tilde{p}\left(Z_{L}-\mu_{L}|X\right)\right]$ at the target spatial-temporal blocks.

Next, we study prediction of the latent process at spatial-temporal blocks. Based on monthly data available at the 100 locations, our aim is to predict quarterly aggregated data in a collection of spatial blocks, instead of points. Here, a quarter corresponds to consecutive three-month periods, as shown by alternating shaded regions in Figure 5A. We divide the spatial domain, the unit square [0, 1]^2^, for the four quarters into 40, 50, 30, and 60 irregular spatial blocks, respectively, obtained by a Voronoi tessellation based on the same number of randomly sampled points, using the R package deldir (Turner 2024). Given the simulated data, we seek the posterior predictive distribution of the aggregated latent process quarterly in these spatial blocks throughout the quarters, based on the stacked posterior $\tilde{p}\left(Z_{L}|X\right)$. We assume that the mean function μ(⋅) is characterized by the monthly indicator basis function $\psi^{\left(1\right)}$, as defined in (19). Figure 6 compares the quarterly aggregated true detrended spatial-temporal surface Z(ℓ)-μ(ℓ), with which the data were simulated, and its corresponding posterior median in the spatial blocks.

We notice that the block-level posterior distributions closely reflect the true spatial-temporal pattern, indicating that our proposed model accurately captures the underlying process. This further demonstrates the flexibility of our proposed hierarchical model (4), as it accommodates complicated survey designs with time-varying spatial blocks.

### 5.4 | Adjusting for Unmeasured Spatial-Temporal Confounding

Approaches to mitigating spatial confounding include Restricted Spatial Regression (RSR), which restricts spatial random effects to the subspace orthogonal to the column space of the covariates (Hodges and Reich 2010). In our simulation study, we therefore compare three strategies for estimating the fixed effects: (i) a baseline model that ignores unmeasured confounding, (ii) a model that augments the regression with spatio-temporal basis functions evaluated at the centroids of the blocks, and (iii) a modular RSR in which the basis functions are adjusted with respect to the covariates. Because our framework integrates inference over the posterior distribution of the latent predictor $Z\left(L_{k}\right)$, this constitutes a modular Bayesian approach. Consequently, unlike classical RSR which performs a single orthogonalization with respect to the observed covariates, we orthogonalize the basis functions iteratively for each posterior sample of $Z\left(L_{k}\right)$, ensuring that the adjustment is coherent with the modular structure. Although RSR has been noted to potentially degrade prediction and produce unstable inference in some settings (Zimmerman and Hoef 2022; Khan and Calder 2022), including it in our simulation allows us to directly evaluate its behavior relative to the basis-only approach using the Bayesian MSE of the fixed effects.

We construct the basis as a tensor-product of Gaussian radial basis functions (RBFs) in both space and time, given by $\zeta_{j,m}(s,t)=\xi_{j}(s)\times \eta_{m}(t)$, where $\xi_{j}(s)$ and $\eta_{m}(t)$ denote the spatial and temporal components, respectively. The only choices required are the number of spatial basis functions $K_{s}$ and the number of temporal basis functions $K_{t}$; all scales are fixed a priori and only the corresponding coefficients are estimated. For the spatial component, we select $K_{s}$ knots $\kappa_{1},\;\ldots ,\kappa_{K_{s}}$ via k-means clustering on the centroids of the spatial blocks and define $\xi_{j}(s)=exp\left(-{\left\|s-\kappa_{j}\right\|}^{2}/r_{1}^{2}\right)$, where $r_{1}$ is fixed heuristically, for example as the median pairwise distance between the centroids. This produces smooth but not overly diffuse spatial features, and no spatial smoothness parameter is estimated. For the temporal component, we select $K_{t}$ knots $\tau_{1},\;\ldots ,\tau_{K_{t}}$ evenly spaced over the domain defined by the temporal blocks and define $\eta_{m}(t)=exp(-{\left|t-\tau_{m}\right|}^{2}/r_{2}^{2})$, where $r_{2}$ is also assumed fixed as the median pairwise distance between knots. Then this modifies the regression model in (4) to

(20) $$Y\left(L_{k}\right)=w{\left(L_{k}\right)}^{⊤}\beta_{1}+\beta_{2}Z\left(L_{k}\right)+\sum_{j=1}^{K_{s}}\sum_{m=1}^{K_{t}}\beta_{j,m}\xi_{j}\left(c_{k}^{s}\right)\eta_{m}\left(c_{k}^{t}\right)+\epsilon_{k},$$

where $c_{k}^{s}$ denote the centroids of the spatial component and $c_{k}^{t}$ denote the midpoint of the temporal component of the spatial-temporal block $L_{k}$ for each k.

We find from Table 1 that across all levels of confounding (ρ), incorporating spatial-temporal basis functions consistently reduces the Bayesian MSE for the fixed effects compared with the unadjusted linear model, indicating improved robustness to unmeasured confounding. The modular RSR performs similarly to the basis-only approach but offers no substantial additional gains, particularly for the covariates $w_{1}$ and $w_{2}$. For the coefficient of Z(L), the gains are modest but still show a slight reduction in error when using basis adjustments, supporting the usefulness of flexible spatial-temporal basis functions in mitigating confounding.

### 5.5 | Model Comparison

We evaluate and compare the predictive performance of our proposed model against some alternative approaches. For alternative models, we regress the result $Y\left(L_{k}\right)$ for each k, as defined in (4), on the predictors $w\left(L_{k}\right)$ and $X\left(L_{k}\right)$. Here, $X\left(L_{k}\right)$ denotes the value of the covariate aggregated in the spatial-temporal block $L_{k}$ and is distinct from the latent process $Z\left(L_{k}\right)$. We use available off-the-shelf spatial interpolation tools to estimate $X\left(L_{k}\right)$. More specifically, we consider two competing approaches—multilevel B-spline approximation (MBA), implemented using the mba.surf() function from the R package MBA (Finley et al. 2024), and, spatial kriging (kriging) using the krige.conv() from the R package geoR (Ribeiro Jr et al. 2024). Unlike our proposed method, MBA and kriging proceed by first obtaining point estimates in a fine grid in the spatial domain and then calculating averages in each spatial block, completely ignoring the uncertainty surrounding the estimation procedure. In addition, none of the alternative methods account for temporal correlation in the observed data, and temporal aggregation at the quarterly level is achieved by simply averaging over monthly observations.

The predictive accuracy for each model is measured using the widely applicable information criterion, WAIC (Watanabe 2010; Gelman et al. 2013). We evaluated WAIC for each model on synthetic data. From Table 2, we notice that all methods deliver similar predictive performance, with our proposed method slightly better. In Table 2, we also report the mean squared error (MSE) for block-level predictions of the spatial–temporal process. The MSEs are similar across methods, with stacking showing a slight increase because its MSE is computed using samples from the stacked posterior and therefore reflects full parameter uncertainty, whereas the kriging and MBA MSEs rely on plug-in predictions that do not account for such uncertainty. We also note that these MSEs are not strictly comparable, as the stacking MSE is a Bayesian measure while the kriging and MBA MSEs are frequentist, reflecting fundamentally different sources of uncertainty.

Moreover, the execution times for both MBA and kriging depends on the grid resolution. Also, contrary to common assumption, increasing the grid resolution has little impact on predictive accuracy, probably because finer resolution spatial interpolation contributes little when they are aggregated to obtain block-level estimates. Executing the stacking algorithm corresponds to fitting 54 candidate models in parallel across 6 cores. Hence, besides offering competitive predictive performance with reasonable run-time, a crucial advantage of our framework over other methods is the ability to deliver fully model-based uncertainty quantification for the latent spatial-temporal process at any arbitrary location and time.

## 6 | Data Analysis

**FIGURE 7 |:**
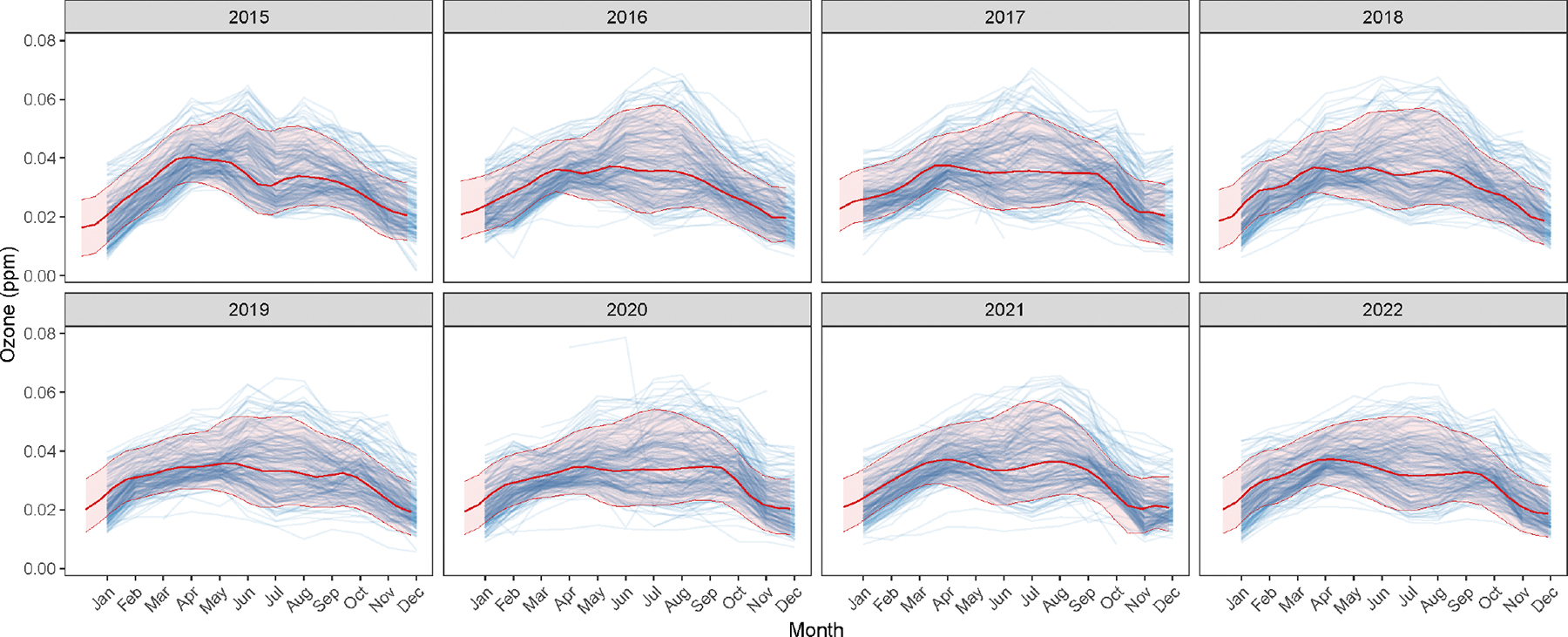
95% credible intervals (in *red*) of ozone concentration predictions obtained from the stacked posterior $\tilde{p}(Z(\ell )|X$, using a periodic Fourier basis mean.

We study the effects of ozone concentration on asthma-related ED visits by first estimating the spatial-temporal regression module based on available monthly ozone concentration data. We use the periodic Fourier basis function, as given by $\psi^{\left(2\right)}(\cdot )$ in (19). Guided by visual inspection of the temporal trends in ozone measurements (see Figure S4 of the Supporting Information), we consider periods of 5, 6, 7, and 12. We consider the separable spatial-temporal covariance kernel (12). For candidate values of ϕ, we choose $G_{\phi_{1}}=\{0.3,\;0.5,\;1\}$, $G_{v}=\{0.5,\;1,\;1.5\}$, and $G_{\delta^{2}}=\{1.5,\;2\}$. Hence, we stack on the 54 models obtained by the Cartesian product of each of these grids. Once we obtain optimal stacking weights based on 1000 posterior samples of $\left\{Z_{\tilde{\ell }},\gamma ,\sigma^{2}\right\}$, we perform posterior predictive inference for ozone concentrations at 20,000 unique space-time coordinates, comprising 100 randomly sampled locations within the convex hull of monitoring sites at 200 time points spanning 2015–2022. Figure 7 displays the 95% posterior predictive credible intervals for the latent spatial-temporal process at these space-time locations, overlaid on the observed ozone measurements. The uncertainty band is estimated from the stacked posterior $\tilde{p}(Z(\ell )|X)$. We notice that the temporal trend is captured reasonably well.

**FIGURE 8 |:**
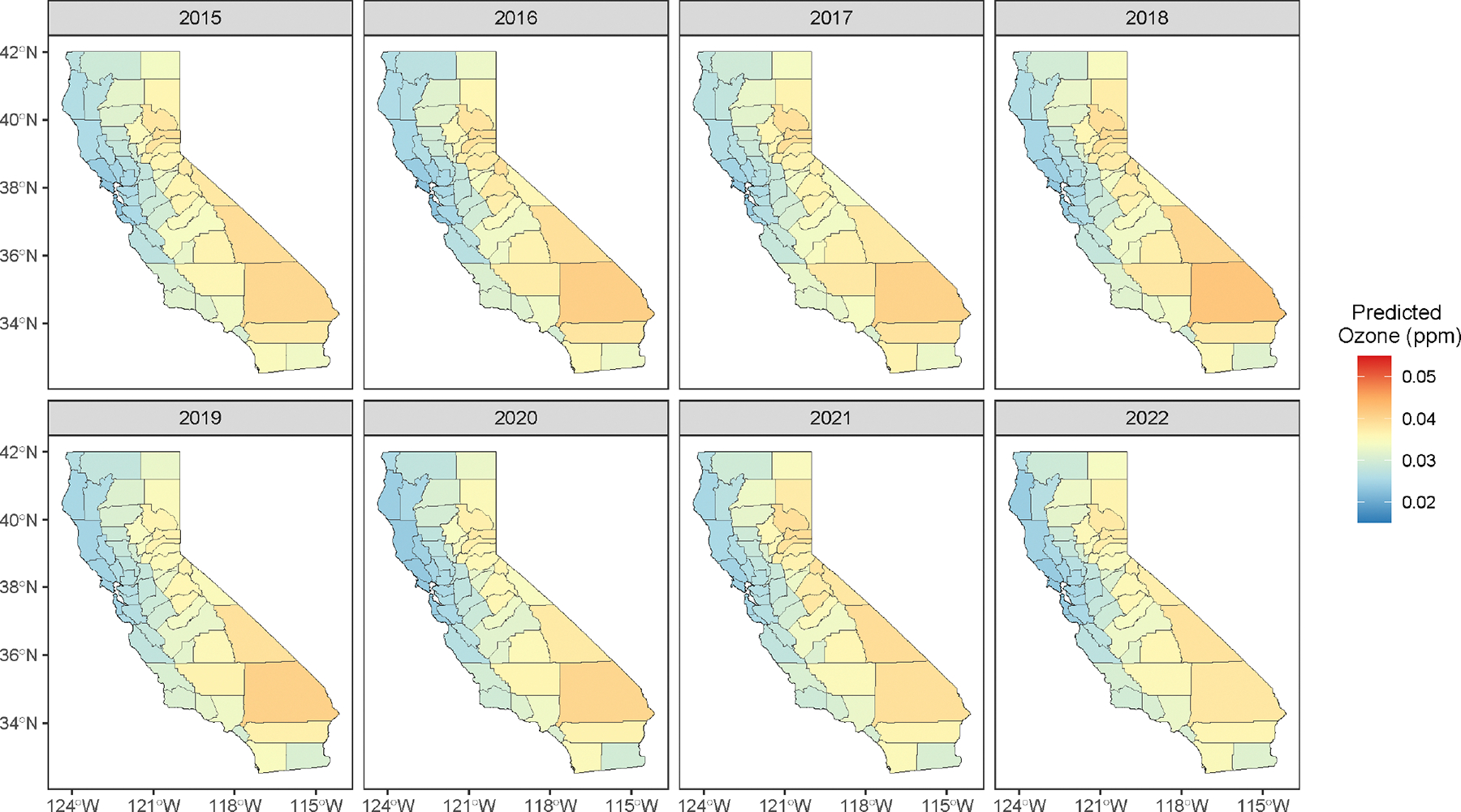
Posterior median of annual ozone concentration predictions at counties of California, obtained from monthly point-referenced observations.

Next, we obtain annual county-level ozone concentration predictions based on the stacked posterior $\tilde{p}(Z(L)|X)$. Figure 8 shows the posterior median of annual ozone concentration predictions in each county. The spatial patterns closely resemble those in Figure 3, which shows interpolated surfaces from aggregated monthly monitoring data. However, the pronounced ozone hotspots (in the counties of Riverside, San Bernadino, Inyo, Sierra, Plumas, El Dorado) and the low-concentration regions (e.g., Humboldt, Mendocino, Sonoma, Marin, San Francisco, San Mateo, Santa Cruz) in Figure 3 are somewhat less pronounced in their corresponding counties in Figure 8. As anticipated, spatial averaging dampens local fluctuations, resulting in smoother estimates that may blur sharp variations in the observed data; nevertheless, the overall pattern of higher inland regions remains largely preserved. We use these posterior predictive samples of annual county-level ozone concentrations to estimate its effect on the asthma-related ED visits.

Subsequently, we estimate a linear regression module with the objective of studying the effect of ozone, race/ethnicity, and time, on age-adjusted asthma-related emergency department visit rates. For clarity, we reformulate the linear model in (4) using symbolic notation as follows. Suppose $Y_{ijt}$ denotes the age-adjusted asthma-related ED visit rate (per 10,000) for county i, race/ethnicity j and year t, for i=1,…58,j=1,…,5 and t=1,…,8. We consider the log-linear model

(21) $$\begin{array}{l} log\left(Y_{ijt}\right)=\beta_{0}+\beta_{1}\cdot {\text{Race}}_{j}+\beta_{2}\cdot {\text{Ozone}}_{it}+\beta_{3}\cdot {\text{Year}}_{t}\\ \;+\;(\text{Basis}{)}_{it}+\epsilon_{ijt}\;, \end{array}$$

where independent errors $\epsilon_{ijt}\sim \;N(0,{\left|B_{i}\right|}^{-1}\tau^{2})$, and ${\left(Basis\right)}_{it}$ is constructed analogously as (20) with $K_{s}=20$ and $K_{t}=1$, capturing unmeasured spatial confounding. We omit the temporal component in the basis since Year is already present in the model. Moreover, $\beta_{0}$ is the intercept, ${Race}_{j}$ is a 4 × 1 dummy-encoded vector representing the j th racial group, $\beta_{1}={\left(\beta_{11},\beta_{12},\beta_{13},\beta_{14}\right)}^{⊤}$ is the corresponding 4 × 1 vector of regression coefficients, ${Ozone}_{\text{it}}$ is the estimated ozone concentration for county i in year $t,\;{Year}_{t}=t$ for each $t,\left|B_{i}\right|$ is the area of county i, and $\epsilon_{ijt}$ denote independent measurement errors. We assume a hierarchical model surrounding (21) as given by (4). We assume the Gaussian prior $\beta |\tau^{2}\sim N\left(0,\tau^{2}V_{\beta }\right)$ with $V_{\beta }=10^{3}I_{7}$, where $\beta ={\left(\beta_{0},\beta_{1}^{⊤},\beta_{2},\beta_{3}\right)}^{⊤}$, and place an inverse gamma prior $\tau^{2}\sim IG(0.01,\;0.01)$.

**FIGURE 9 |:**
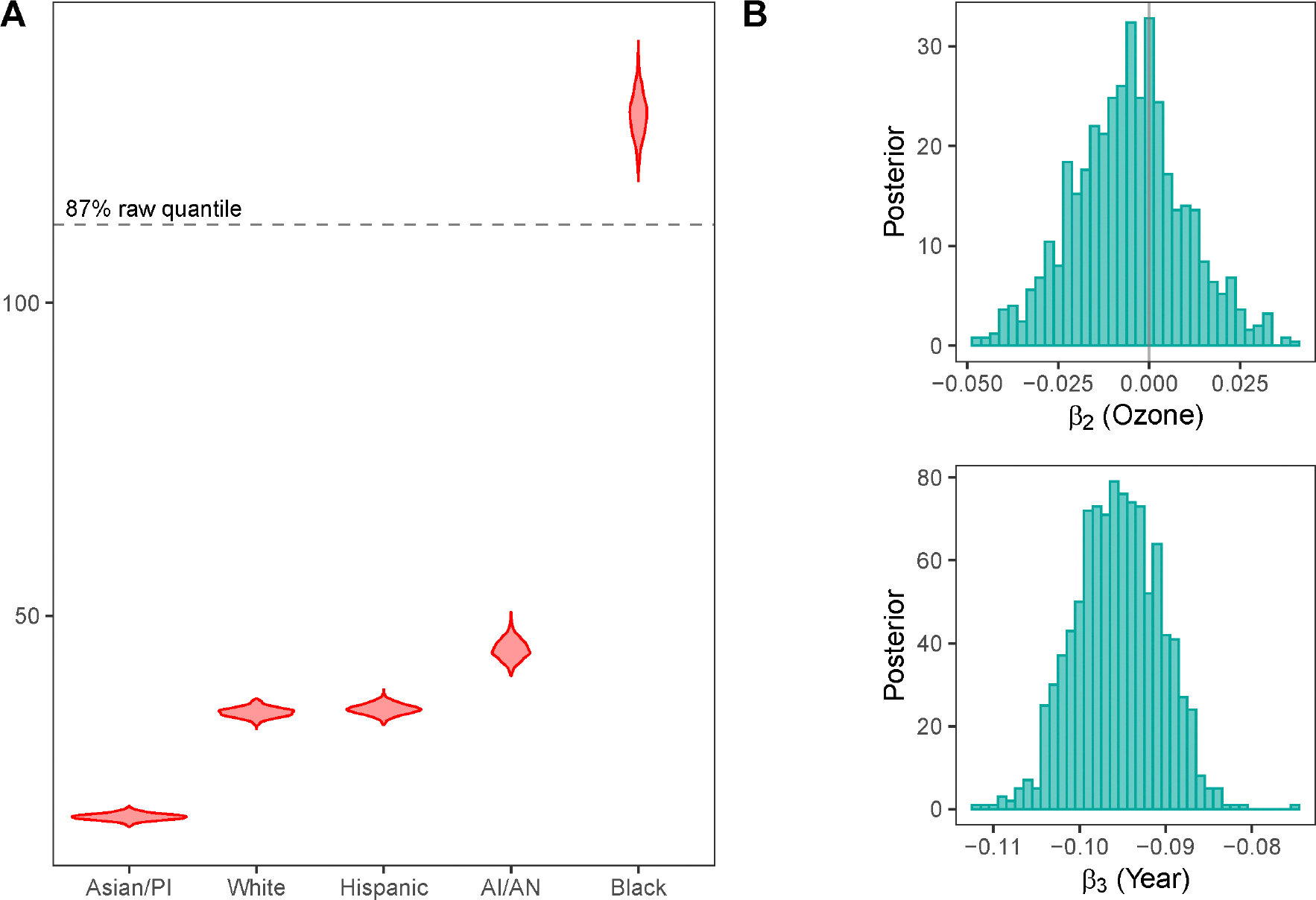
A: Posterior distributions of relative effect sizes at constant ozone concentration and year; AI/AN: American Indian/Alaskan native, PI: Pacific Islanders. B: Posterior distributions of the coefficients corresponding to ozone and year.

We present a comprehensive summary of the posterior distributions of the regression coefficients in Table 3. We find that all regression coefficients except $\beta_{14}$ have 95% posterior credible intervals that exclude zero, suggesting strong evidence of meaningful differences in contributions specific to each racial group, except that the Hispanic and White groups do not differ significantly in their effects on asthma-related health emergencies, for a given year and ozone level. For better understanding, we examine the relative effect sizes corresponding to the racial groups of white ($e^{\beta_{0}}$), American Indian/Alaskan native ($e^{\beta_{0}+\beta_{11}}$), Asian/Pacific Islander ($e^{\beta_{0}+\beta_{12}}$), black ($e^{\beta_{0}+\beta_{13}}$), and Hispanic ($e^{\beta_{0}+\beta_{14}}$) racial groups, which reflects the expected rates of asthma-related ED visits for each racial group in the reference year 2015, when exposed to the baseline level of ozone of 0.03 ppm. Figure 9A illustrates the posterior distributions of the relative effects sizes of each racial group.

Our analysis reveals that the average asthma-related ED visit rates of the black group are approximately 3.7 times the average rates of the white (CI: 3.5, 4) and Hispanic (CI: 3.5, 3.9) groups, 2.9 (CI: 2.7, 3.1) times the average rate of the American Indian/Alaskan native group, and around 7.3 (CI: 6.8, 7.8) times the average rate of the Asian/Pacific Islander group, when all are exposed to the same levels of ozone concentration, during the year 2015. Here, the reported values represent posterior medians, and “CI” denotes the corresponding 95% posterior credible intervals. Moreover, as seen from Figure 9A, the relative effect size of the black group lies above the 87th percentile of the observed rates. These substantial differences highlight the persistent and disproportionate burden of asthma-related emergency department visits among the black population in California.

Figure 9B displays the posterior distributions of the regression coefficients corresponding to ozone concentration and year. We observe a marginally negative association between ozone and asthma-related ED visit rates. This suggests a weak inverse relationship, with higher ozone levels associated with slightly lower asthma-related ED visit rates. Although the association is not statistically strongly supported, it is consistent with patterns reported in previous studies (Zhu et al. 2003). This also contrasts with the commonly held assumption that a higher concentration of ozone increases the risk of asthma-related symptoms. More specifically, we find that an increase of 0.005 ppm of ozone concentration leads to a drop in asthma-related ED visit rates by a factor of 0.99 (CI: 0.96, 1). This relationship suggests seasonal interactions of ozone (Quick et al. 2015). Ozone levels also tend to be higher in rural inland areas (e.g., Central Valley), population density is lower, and ED utilization is lower due to access barriers, which does not necessarily imply a lower incidence of asthma. Urban coastal areas (e.g., San Francisco Bay area, Los Angeles) may have lower ozone but higher asthma rates due to other pollutants, higher population density and reporting, and different healthcare-seeking behaviors. Our analysis shows that each year is associated with a 10% annual decrease in asthma-related ED visit rates, with the expected rate decreasing by a factor of 0.91 (CI: 0.90, 0.92). This is consistent with the downward trend in Figure S2 of the Supporting Information.

We fit the model on 15,725 space-time coordinates using a stacked posterior that combines 54 candidate models. The entire procedure took roughly 90 min, a considerable improvement over traditional full Bayesian MCMC approaches, where a single iteration could take over 15 min. Furthermore, our regression inference framework accounts for the uncertainty in the estimated ozone concentrations by integrating the samples from their posterior distribution. This ensures that both the latent process and its downstream effects are quantified in a fully probabilistic and computationally efficient manner. Moreover, the use of periodic Fourier basis functions for modeling temporal trends enables smooth interpolation and prediction at any time point within the study window. This flexibility would be difficult to achieve using conventional discrete-time models. In addition, a model relying on monthly basis functions would be unable to provide predictions for months with no available data, which is a likely scenario given the irregularity common in environmental monitoring records.

## 7 | Discussion

A key strength of our modular Bayesian framework is its capacity to jointly estimate ozone concentrations at arbitrary spatial and temporal resolutions, along with their association with an outcome of interest. Stacking of predictive densities enables us to obtain fully model-based uncertainty quantification for all model parameters by averaging over a collection of candidate models, each representing different specifications of process parameters. Future methodological directions may involve developing a multivariate areal time series model in place of the current linear regression framework, with the goal of capturing additional sources of variability and complex temporal-spatial dependencies inherent in the data. The data analysis presented in this article not only underscores existing racial disparities in health outcomes but also points to systemic inequities in environmental exposure, access to healthcare, and underlying social determinants of health. It emphasizes the need for targeted public health interventions and policies that address structural drivers of asthma morbidity and improve health equity in racial and ethnic communities. Future analyses may consider jointly estimating multiple exposure variables, such as NO_2_, PM_2.5_, and others, using multivariate spatial-temporal regression models to better account for potential correlations among pollutants and their combined effects on health outcomes.

## Supplementary Material

Supplement

Supporting Information

Additional supporting information can be found online in the Supporting Information section. **Data S1.** Supporting Information.

## Figures and Tables

**TABLE 1 |:** Bayesian MSE of the fixed-effects under varying levels of spatial–temporal confounding (*ρ*), comparing different approaches within a modular Bayesian framework, based on 50 replications.

		*ρ*		
Coefficient	Method	0.1	0.5	0.9

*𝑤*_1_	modularLM	12.95	12.46	11.81
	modularLM+Basis	12.51	12.07	11.51
	modularLM+RSR	12.84	12.31	11.62
*𝑤*_2_	modularLM	0.48	0.47	0.46
	modularLM+Basis	0.47	0.46	0.45
	modularLM+RSR	0.47	0.46	0.46
*Z*(*L*)	modularLM	0.64	0.59	0.55
	modularLM+Basis	0.61	0.57	0.53
	modularLM+RSR	0.64	0.59	0.54

**TABLE 2 |:** Comparison of the predictive performance of our proposed method based on predictive stacking against alternative approaches, based on 50 replications.

Method	Model-based UQ^a^	Temporal dependence	*n*_grid_^b^	MSE	WAIC	Run Time (in secs)

MBA	**✗**	**✗**	50	0.61	1714.84	18.10
			100	0.60	1714.82	73.82
			200	0.60	1714.79	265.54
kriging	**✗**	**✗**	50	0.60	1714.74	19.57
			100	0.59	1714.73	85.32
			200	0.59	1714.73	320.91
Stacking	**✓**	**✓**	—	0.63	1714.52	257.55

a UQ: Uncertainty quantification.

b *n*_grid_: axis resolution for spatial interpolation used in block-level estimation.

**TABLE 3 |:** Posterior summary of regression coefficients from the hierarchical regression (21) relating asthma-related emergency department visit rates in logarithmic scale to race indicators, year, and predicted county-level average ozone concentration, adjusted for unmeasured spatial confounding.

Parameter	Effect	Posterior median	95% credible interval	Details

*β*_0_	Intercept	3.54	(3.49, 3.59)	
	*Reference group*: White			
*β*_11_	American Indian/Alaskan native	0.25	(0.18, 0.33)	
*β*_12_	Asian/Pacific Islander	−0.66	(−0.73, −0.59)	
*β*_13_	Black	1.33	(1.27, 1.39)	
*β*_14_	Hispanic	0.02	(−0.04, 0.07)	
*β*_2_	Ozone (per 0.005 ppm)	−0.01	(−0.04, 0.02)	*baseline*: 0.03 ppm
*β*_3_	Year	−0.09	(−0.10, −0.09)	*baseline*: 2015
*τ* ^2^	Error variance	0.12	(0.11, 0.13)	

## Data Availability

The asthma data is openly available at the CalHHS website hosted by the California Department of Public Health (CDPH). The data on ozone measurements and geographic locations of the air quality monitoring stations are collected using the Air Quality and Meteorological Information System (AQMIS) database query tool of the California Air Resource Board (CARB). Supplementary code to reproduce the findings presented in this article can be found at https://github.com/SPan-18/AsthmaOzoneCA. The developed code is bundled into an R package, named spStackCOS, and can be installed from https://github.com/SPan-18/spStackCOS-dev.
