# Supplementary material for: Bayesian Inference for Spatially-Temporally Misaligned Data Using Predictive Stacking
Source: Environmetrics. Author manuscript; Available in PMC 2026 May 1. (PMC13129565; doi:10.1002/env.70072)
Supplement: Supplement [file NIHMS2163847-supplement-Supplement.pdf]

SUPPLEMENTARY MATERIALS FOR "BAYESIAN INFERENCE FOR SPATIALLY-TEMPORALLY MISALIGNED DATA USING PREDICTIVE STACKING"

SOUMYAKANTI PAN* AND SUDIPTO BANERJEE*

*Department of Biostatistics, University of California Los Angeles

S1. Descriptive overview of the dataset

In this section, we present an exploratory data analysis of age-adjusted emergency department (ED) visit rates per 10,000 residents in California, across counties and racial groups. Figure S1 display annual county-level ED visit rates for each racial group throughout the study period 2015-22, revealing clear spatial and racial patterns. Notably, the black population consistently reports higher ED visit rates across the study period, with elevated rates concentrated in the coastal areas of northern California. In terms of data completeness, the white racial group has 2.1% missing data and the black group has 10.8% missing data, while the Asian/Pacific Islander and American Indian/Alaska Native groups exhibit high levels of missingness, with 34.1% and 59.3% missing respectively. On the other hand, at the county level, Los Angeles, Riverside, Sacramento and San Diego counties have complete ED visit data. The counties with the most missing data are Lassen, Siskiyou, Calaveras, Del Norte, Plumas, Mariposa, Modoc, Glenn, Nevada, and Trinity, each exhibiting approximately 50–60% missingness. These counties are predominantly rural and located in Northern California and the Sierra Nevada region. These areas are characterized by mountainous terrain and lower population density, which may contribute to challenges in healthcare access and data reporting completeness. These patterns underscore the importance of considering both spatial and demographic dimensions in analyzing the data.

Next, we plot the ED visit rates over time to examine temporal trends within each racial group. As shown in Figure S2, most groups exhibit a gradual decline in rates over the study period, suggesting potential improvements in underlying health conditions, access to preventive care, or changes in reporting and healthcare utilization. A strong declining trend is particularly evident for the white and the Asian/Pacific Islander groups while other groups show more variability. These patterns provide important context for interpreting cross-sectional differences and support the inclusion of temporal components in our modeling framework.

Next, we provide justification for the Gaussian modeling assumptions on the log-transformed ED visit rates. As shown in Figure S3A, the distribution of the raw ED visit rates exhibits strong positive skewness across all racial groups, with a long right tail driven by a small number of counties reporting exceptionally high rates. This pronounced skewness is inconsistent with the

E-mail address: span18@ucla.edu, sudipto@ucla.edu.

symmetry assumed under Gaussian models. Applying a log transformation helps stabilize the variance and mitigate the effect of extreme values, resulting in a distribution that is more symmetric and approximately Gaussian (see Figure S3B). The log-transformed rates exhibit reduced variability across the range of values and are more amenable to modeling under Gaussian assumptions.

Further, we plot the recorded measurements of ozone levels across time and observe clear seasonal fluctuations and periodic patterns, indicative of strong temporal structure (see Figure S4). These recurring trends suggest the need to account for seasonality when modeling the temporal dynamics of ozone exposure. In addition to temporal variation, we also find substantial spatial heterogeneity in ozone concentrations, highlighting the importance of incorporating spatial effects into the modeling framework. Together, these observations motivate the use of flexible spatial-temporal models that can effectively capture both the periodic temporal behavior and the variability across space.

S2. Additional simulation results

Calculation of leave-one-out predictive densities is central to evaluating the objective function used to determine optimal stacking weights. In the main article, we present two approaches for computing LOO predictive densities. The first is an exact method based on closed-form expressions, while the second is an approximate method known as Pareto Smoothed Importance Sampling (PSIS). To assess the accuracy of PSIS relative to the exact method, we conduct a simulation study using a spatial regression model. Suppose $\{s_1, \ldots, s_n\}$ denote a collection of n locations in the unit square, where we simulate spatially point-referenced responses $X(s_i)$ for $i = 1, \ldots, n$, using

$$X(s_i) = W\beta + Z(s_i) + \epsilon(s_i) \ , \quad \epsilon(s_i) \overset{\text{ind}}{\sim} \mathsf{N}(0, \delta^2\sigma^2) \ , \tag{S1}$$

where W is $n \times 2$ comprising of an intercept and a predictor sampled from a standard normal distribution, $\beta = (2, 5)^\top$, $\epsilon(s_i)$ for each i denote independent measurement error, $Z(s_i)$ are realizations of central spatial Gaussian process, given by $Z(s) \sim \mathsf{GP}(0, C_s(s, s'; \phi_1, \nu))$, with covariance function $C_s(\cdot, \cdot)$ as defined in (12) of the main article. We take $\sigma^2 = 0.4$, $\phi_1 = 2$, $\nu = 0.5$ and $\delta^2 = 1.5$.

We assign priors to β and σ^2 as discussed in Section 4.1 of the main article. We fix the values of $\phi_1 = 3.5$, $\nu = 0.75$, and $\delta^2 = 1$. Under this model and prior specification, we compute the exact leave-one-out predictive densities using closed-form expressions. To obtain the leave-one-out predictive densities via PSIS, we use posterior samples of β and $Z = (Z(s_1), \ldots, Z(s_n))^\top$ obtained using the R package `spStack` (Pan and Banerjee, 2024), and calculate the log-pointwise predictive densities (lppd). We then apply the `psis()` function from the R package `loo` (Vehtari et al., 2024) to compute the leave-one-out predictive densities using stabilized importance weights. Figure S5 presents a comparison of the two methods, showing no visible differences in the resulting predictive densities. However, the exact method is considerably more computationally intensive than PSIS, demonstrating the latter's efficiency for large datasets.

S3. Technical details

Proposition 1. *For any $a < b$ and $c < d$, suppose $\tilde{C}_t(a, b, c, d; \phi) = \int_c^d \int_a^b C_t(t, t'; \phi_2) dt dt'$, then*

(a) *for non-overlapping* (a,b) *and* (c,d)*, with* $a < b \leq c < d$*,*

$$\tilde{C}_t(a,b,c,d;\phi_2) = \frac{1}{\phi_2^2}\left[F(a,d) + F(b,c) - F(a,c) - F(b,d)\right] \ ,$$

(b) *if intervals* (a,b) *and* (c,d) *overlap, with* $a \leq c < b \leq d$*,*

$$\tilde{C}_t(a,b,c,d;\phi_2) = \frac{1}{\phi_2^2}\left[2\phi_2(b-c) + F(a,d) + F(c,b) - F(a,c) - F(b,d)\right] \ ,$$

(c) *if* (a,b) *is nested within* (c,d)*, i.e. either* $c \leq a < b < d$ *or* $c < a < b \leq d$*,*

$$\tilde{C}_t(a,b,c,d;\phi_2) = \frac{1}{\phi_2^2}\left[2\phi_2(b-a) + F(a,d) + F(c,b) - F(c,a) - F(b,d)\right] \ ,$$

where $F(a_1,a_2) = F(a_1,a_2;\phi_2) = \exp\left(-\phi_2(a_2-a_1)\right)$ *for any* $a_1,a_2 \in \mathcal{T}$*.*

Proof. (a) Without loss of generality, we assume $a < b < c < d$. The condition $a < b < c < d$ denotes that the intervals (a,b) and (c,d) are disjoint and (a,b) lies on the left of (c,d). Since, $t \in (a,b)$ and $t' \in (c,d)$. This means $t' \geq t$ holds true always, and hence, $|t-t'| = t'-t$. So, we evaluate the integral I_1 as follows.

$$\begin{aligned}
I_1 &= \int_c^d \int_a^b \exp(-\phi_2|t-t'|)dtdt' = \int_c^d \int_a^b \exp(-\phi_2(t'-t))dtdt' \\
&= \frac{1}{\phi_2}\left(e^{\phi_2 b} - e^{\phi_2 a}\right)\int_c^d \exp(-\phi_2 t')dt' = \frac{1}{\phi_2^2}\left(e^{\phi_2 b} - e^{\phi_2 a}\right)\left(e^{-\phi_2 c} - e^{-\phi_2 d}\right) \\
&= \frac{1}{\phi_2^2}\left(e^{\phi_2(b-c)} - e^{\phi_2(b-d)} - e^{\phi_2(a-c)} + e^{\phi_2(a-d)}\right) \\
&= \frac{1}{\phi_2^2}\left[F(b,c) - F(a,c) - F(b,d) + F(a,d)\right]
\end{aligned}$$

The equality case $b = c$ simply implies $F(c,b) = 0$.

(b1) If $c = a$ and $d = b$, then we are dealing with the integration

$$\begin{aligned}
\int_a^b \int_a^b e^{-\phi_2|t-t'|} dt dt' &= \int_a^b \left(\int_a^{t'} e^{-\phi_2|t-t'|} dt + \int_{t'}^b e^{-\phi_2|t-t'|} dt \right) dt' \\
&= \int_a^b \left(\int_a^{t'} e^{-\phi_2(t'-t)} dt + \int_{t'}^b e^{-\phi_2(t-t')} dt \right) dt' \\
&= \int_a^b \left(e^{-\phi_2 t'} \cdot \frac{1}{\phi_2} \left(e^{\phi_2 t'} - e^{\phi_2 a} \right) + e^{\phi_2 t'} \frac{1}{\phi_2} \left(e^{-\phi_2 t'} - e^{-\phi_2 b} \right) \right) dt' \\
&= \frac{1}{\phi_2} \int_a^b 1 - e^{\phi_2(a-t')} + 1 - e^{\phi_2(t'-b)} dt' \\
&= \frac{2}{\phi_2}(b-a) - \frac{1}{\phi_2} e^{\phi_2 a} \int_a^b e^{-\phi_2 t'} dt' - \frac{1}{\phi_2} e^{-\phi_2 b} \int_a^b e^{\phi_2 t'} dt' \\
&= \frac{2}{\phi_2}(b-a) - \frac{1}{\phi_2^2} e^{\phi_2 a} \left(e^{-\phi_2 a} - e^{-\phi_2 b} \right) - \frac{1}{\phi_2^2} e^{-\phi_2 b} \left(e^{\phi_2 b} - e^{\phi_2 a} \right) \\
&= \frac{2}{\phi_2}(b-a) - \frac{1}{\phi_2^2} \left(1 - e^{\phi_2(a-b)} + 1 - e^{\phi_2(a-b)} \right) \\
&= \frac{2}{\phi_2^2} \left(\phi_2(b-a) + e^{-\phi_2(b-a)} - 1 \right) \\
&= \frac{2}{\phi_2^2} \left[\phi_2(b-a) + F(a,b) - 1 \right] .
\end{aligned}$$

(b2) In this case, we have $a < c < b < d$. The integration relies on splitting the integral into parts depending on the sign of the term $|t - t'|$, where $t \in (a, b)$ and $t' \in (c, d)$. We split the integral into three double integrals I_1, I_2 and I_3, where I_1 is on $(c, d) \times (a, c)$, I_2 is on $(c, b) \times (c, b)$, and I_3 is on $(b, d) \times (c, b)$.

$$\begin{aligned}
\int_c^d \int_a^b e^{-\phi_2|t-t'|} dt dt' &= \int_c^d \int_a^c e^{-\phi_2(t'-t)} dt dt' + \int_c^b \int_c^b e^{-\phi_2|t-t'|} dt dt' + \int_b^d \int_c^b e^{-\phi_2(t'-t)} dt dt' \\
&= I_1 + I_2 + I_3 ,
\end{aligned}$$

In I_1, $t \le t'$, so we have $|t - t'| = t' - t$. In I_2, $t - t'$ may be both positive and negative. And, for I_3, $t \le t'$, so we have $|t - t'| = t' - t$. We tackle each of these integrals separately.

$$\begin{aligned}
I_1 &= \int_c^d \int_a^c e^{-\phi_2(t'-t)} dt dt' = \int_c^d e^{-\phi_2 t'} \left(\int_a^c e^{\phi_2 t} dt \right) dt' \\
&= \frac{1}{\phi_2} \left(e^{\phi_2 c} - e^{\phi_2 a} \right) \int_c^d e^{-\phi_2 t'} dt' = \frac{1}{\phi_2^2} \left(e^{\phi_2 c} - e^{\phi_2 a} \right) \left(e^{-\phi_2 c} - e^{-\phi_2 d} \right) \\
&= \frac{1}{\phi_2^2} \left(1 - e^{\phi_2(c-d)} - e^{\phi_2(a-c)} + e^{\phi_2(a-d)} \right) .
\end{aligned}$$

Similarly, we find the integral I_3, which is very similar to I_1.

$$\begin{aligned}I_3 &= \int_b^d \int_c^b e^{-\phi_2(t'-t)} dt dt' = \int_b^d e^{-\phi_2 t'} \left(\int_c^b e^{\phi_2 t} dt \right) dt' \\ &= \frac{1}{\phi_2^2} \left(e^{\phi_2 b} - e^{\phi_2 c} \right) \left(e^{-\phi_2 b} - e^{-\phi_2 d} \right) \\ &= \frac{1}{\phi_2^2} \left(1 - e^{\phi_2(b-d)} - e^{\phi_2(c-b)} + e^{\phi_2(c-d)} \right)\end{aligned}$$

Following part (b) of Proposition 1, we have

$$I_2 = \int_c^b \int_c^b e^{-\phi_2|t-t'|} dt dt' = \frac{2}{\phi_2^2} \left(\phi_2(b-c) + e^{-\phi_2(b-c)} - 1 \right)$$

Combining I_1, I_2 and I_3, we have

$$\begin{aligned}I_1 + I_2 + I_3 &= \frac{1}{\phi_2^2} \left(1 - e^{\phi_2(c-d)} - e^{\phi_2(a-c)} + e^{\phi_2(a-d)} \right) + \frac{2}{\phi_2^2} \left(\phi_2(b-c) + e^{-\phi_2(b-c)} - 1 \right) \\ &\quad + \frac{1}{\phi_2^2} \left(1 - e^{\phi_2(b-d)} - e^{\phi_2(c-b)} + e^{\phi_2(c-d)} \right) \\ &= \frac{1}{\phi_2^2} \left(2 - e^{\phi_2(a-c)} + e^{\phi_2(a-d)} - e^{\phi_2(b-d)} - e^{\phi_2(c-b)} \right) \\ &\quad + \frac{2}{\phi_2^2} \left(\phi_2(b-c) + e^{-\phi_2(b-c)} \right) - \frac{2}{\phi_2^2} \\ &= \frac{2}{\phi_2}(b-c) + \frac{1}{\phi_2^2} \left(e^{\phi_2(c-b)} - e^{\phi_2(a-c)} + e^{\phi_2(a-d)} - e^{\phi_2(b-d)} \right) \\ &= \frac{1}{\phi_2^2} \left[2\phi_2(b-c) + e^{-\phi_2(b-c)} - e^{-\phi_2(c-a)} - e^{-\phi_2(d-b)} + e^{-\phi_2(d-a)} \right] \\ &= \frac{1}{\phi_2^2} \left[2\phi_2(b-c) + F(c,b) - F(a,c) - F(b,d) + F(a,d) \right]\end{aligned}$$

(c) In this case the interval (a, b) is nested within the interval (c, d). Hence, we have $c < a < b < d$. We split the integral with respect to t' on (c, d) into three - one over (c, a), one over (a, b) and the last over (b, d). Hence, we have

$$\begin{aligned}\int_c^d \int_a^b e^{-\phi_2|t-t'|} dt dt' &= \int_c^a \int_a^b e^{-\phi_2|t-t'|} dt dt' + \int_a^b \int_a^b e^{-\phi_2|t-t'|} dt dt' + \int_b^d \int_a^b e^{-\phi_2|t-t'|} dt dt' \\ &= I_1 + I_2 + I_3 \ .\end{aligned}$$

We evaluate each of the integrals I_1, I_2 and I_3 separately. First, we find

$$\begin{aligned}I_1 &= \int_c^a \int_a^b e^{-\phi_2(t-t')} dt dt' = \int_c^a e^{\phi_2 t'} \left(\int_a^b e^{-\phi_2 t} dt \right) dt' \\ &= \frac{1}{\phi_2^2} \left(e^{-\phi_2 a} - e^{-\phi_2 b} \right) \left(e^{\phi_2 a} - e^{\phi_2 c} \right) \\ &= \frac{1}{\phi_2^2} \left(1 - e^{\phi_2(c-a)} - e^{\phi_2(a-b)} + e^{\phi_2(c-b)} \right) \ .\end{aligned}$$

Similarly, we find the integral I_3 similar to I_1.

$$
\begin{aligned}
I_3 &= \int_b^d \int_a^b e^{-\phi_2(t'-t)} dt dt' = \int_b^d e^{-\phi_2 t'} \left(\int_a^b e^{\phi_2 t} dt \right) dt' \\
&= \frac{1}{\phi_2^2} \left(e^{\phi_2 b} - e^{\phi_2 a} \right) \left(e^{-\phi_2 b} - e^{-\phi_2 d} \right) \\
&= \frac{1}{\phi_2^2} \left(1 - e^{\phi_2(b-d)} - e^{\phi_2(a-b)} + e^{\phi_2(a-d)} \right)
\end{aligned}
$$

Following part (b) of Proposition 1, we find the integral I_2. Next, we combine I_1, I_2 and I_3.

$$
\begin{aligned}
I_1 + I_2 + I_3 &= \frac{1}{\phi_2^2} \left(1 - e^{\phi_2(c-a)} - e^{\phi_2(a-b)} + e^{\phi_2(c-b)} \right) + \frac{2}{\phi_2^2} \left(\phi_2(b-a) + e^{-\phi_2(b-a)} - 1 \right) \\
&\quad + \frac{1}{\phi_2^2} \left(1 - e^{\phi_2(b-d)} - e^{\phi_2(a-b)} + e^{\phi_2(a-d)} \right) \\
&= \frac{1}{\phi_2^2} \left(2 - 2e^{\phi_2(a-b)} - e^{\phi_2(c-a)} + e^{\phi_2(c-b)} - e^{\phi_2(b-d)} + e^{\phi_2(a-d)} \right) \\
&\quad + \frac{2}{\phi_2}(b-a) + \frac{1}{\phi_2^2} \left(2e^{\phi_2(a-b)} - 2 \right) \\
&= \frac{1}{\phi_2^2} \left[2\phi_2(b-a) + e^{-\phi_2(b-c)} - e^{-\phi_2(a-c)} - e^{-\phi_2(d-b)} + e^{-\phi_2(d-a)} \right] \\
&= \frac{1}{\phi_2^2} \left[2\phi_2(b-a) + F(c,b) - F(c,a) - F(b,d) + F(a,d) \right]
\end{aligned}
$$

□

References

Soumyakanti Pan and Sudipto Banerjee. *spStack: Bayesian Geostatistics Using Predictive Stacking*, 2024. URL https://CRAN.R-project.org/package=spStack. R package version 1.0.1.

Aki Vehtari, Jonah Gabry, Måns Magnusson, Yuling Yao, Paul-Christian Bürkner, Topi Paananen, and Andrew Gelman. loo: Efficient leave-one-out cross-validation and waic for bayesian models, 2024. URL https://mc-stan.org/loo/. R package version 2.8.0.

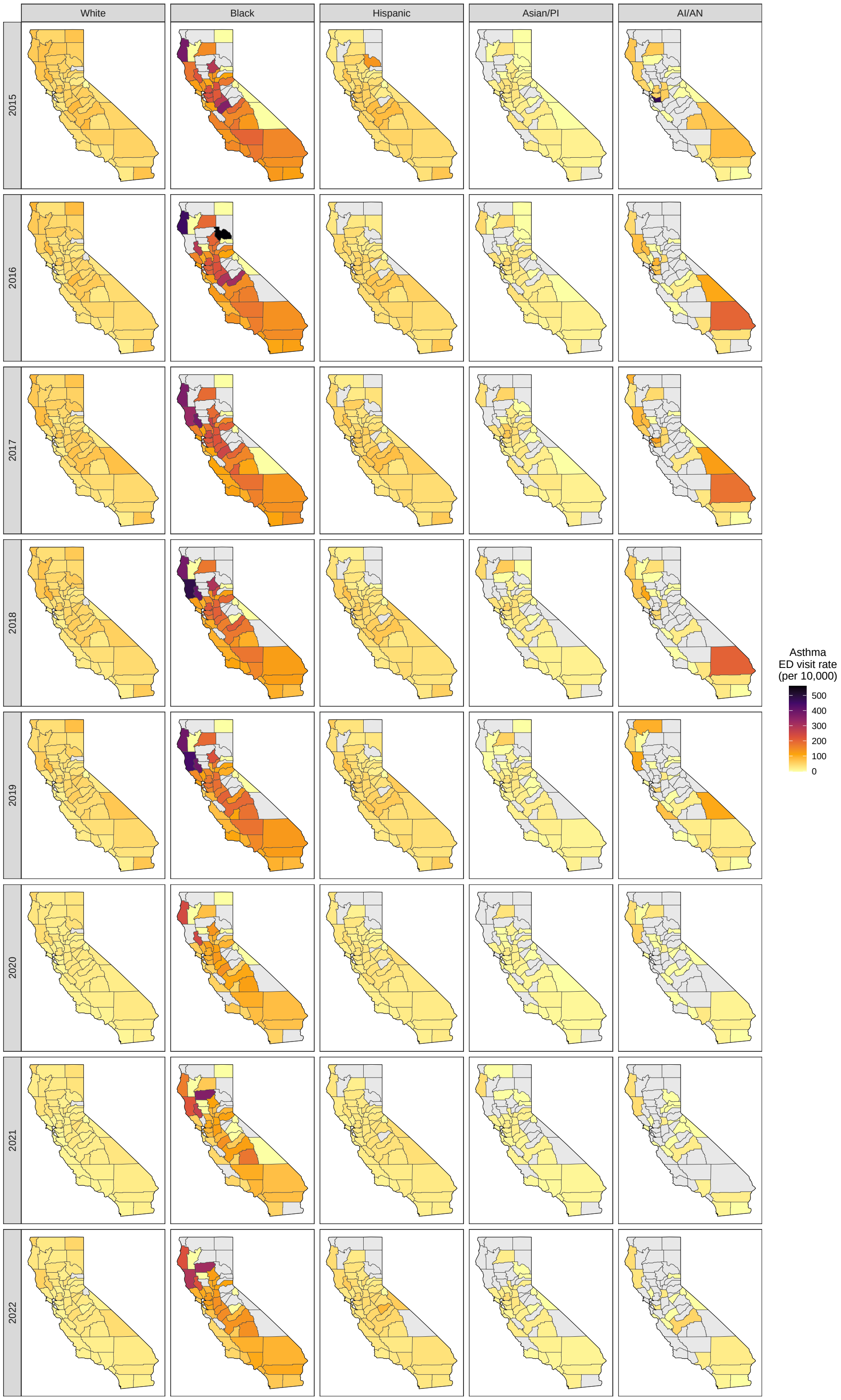

FIGURE S1. Asthma ED visit rates by race 2015-22.

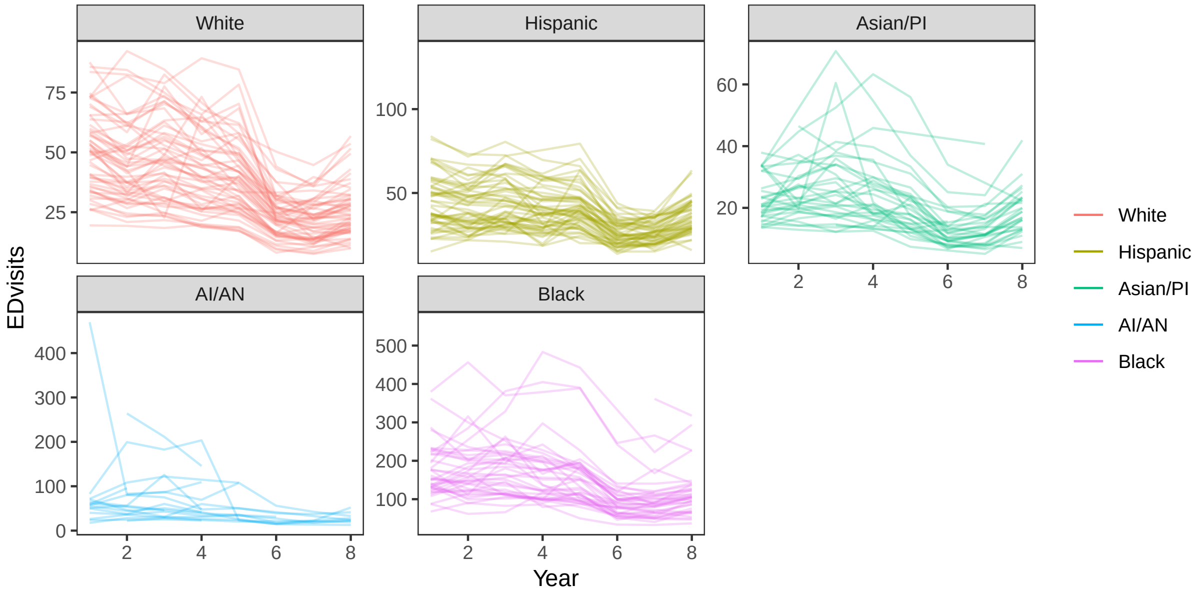

FIGURE S2. Annual trend in asthma-related ED visit rates per 10,000 residents for different racial groups.

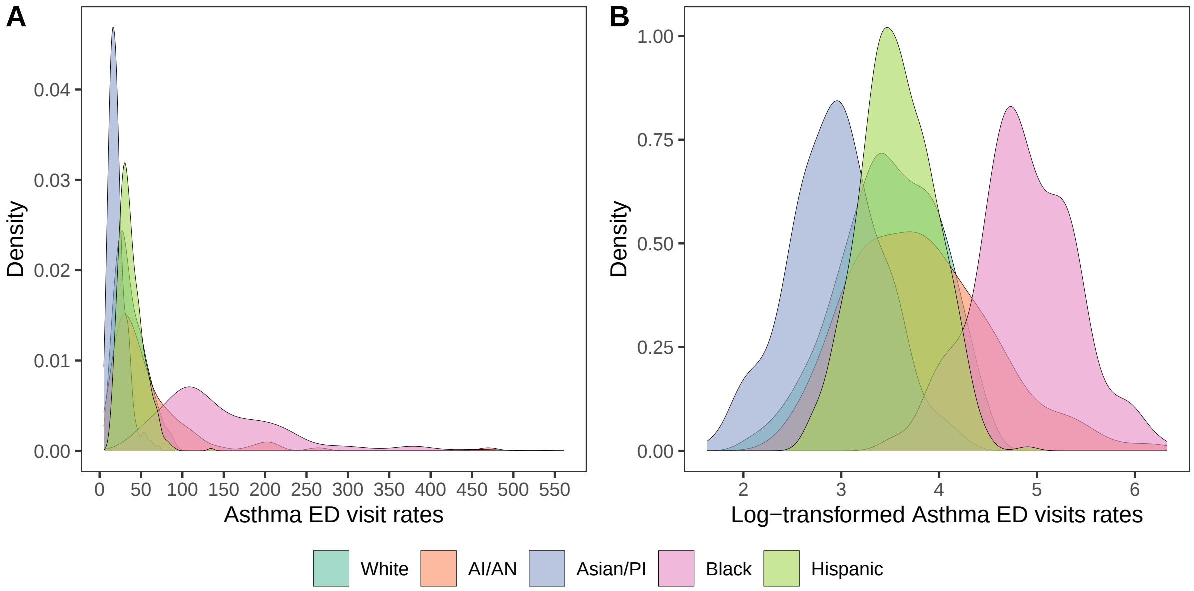

FIGURE S3. Race-specific estimated density of county-level age-adjusted rates (per 10,000) of asthma ED visits during 2015-2022.

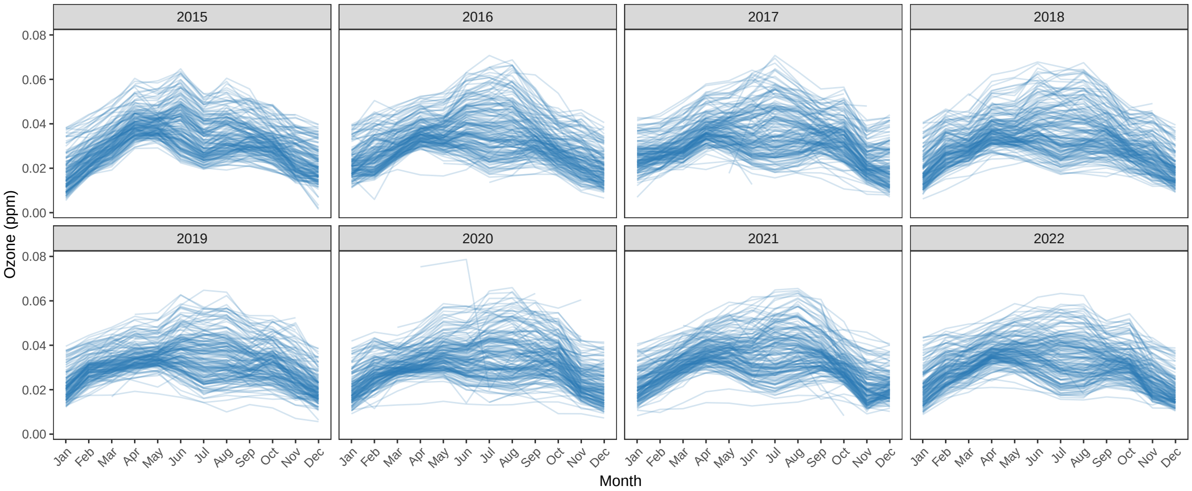

FIGURE S4. Monthly average ozone concentrations (in parts per million, ppm) recorded at various ozone monitoring sites across California from 2015 to 2022. A clear seasonal pattern is evident, with ozone levels peaking as well as exhibiting higher variability during the warmer months (May to October) and reaches troughs during the colder months (November to April).

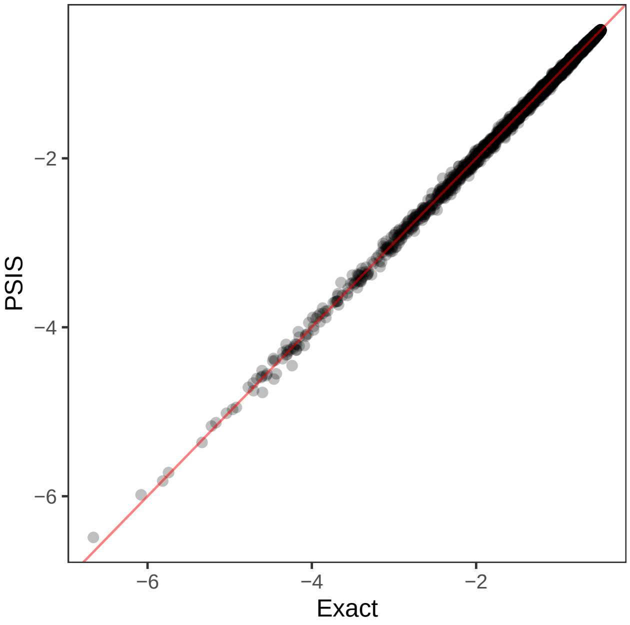

FIGURE S5. Comparison of leave-one-out predictive densities computed using exact closed form expression and Pareto smoothed importance sampling (PSIS) for a spatial regression model on a simulated dataset of sample size 5000.
